# Using uncertainty to link and rank evidence from biomedical literature for model curation

**DOI:** 10.1093/bioinformatics/btx466

**Published:** 2017-07-24

**Authors:** Chrysoula Zerva, Riza Batista-Navarro, Philip Day, Sophia Ananiadou

**Affiliations:** 1 National Centre for Text Mining, School of Computer Science, The University of Manchester, Manchester, UK; 2 Manchester Institute of Biotechnology, The University of Manchester, Manchester, UK

## Abstract

**Motivation:**

In recent years, there has been great progress in the field of automated curation of biomedical networks and models, aided by text mining methods that provide evidence from literature. Such methods must not only extract snippets of text that relate to model interactions, but also be able to contextualize the evidence and provide additional confidence scores for the interaction in question. Although various approaches calculating confidence scores have focused primarily on the quality of the extracted information, there has been little work on exploring the textual uncertainty conveyed by the author. Despite textual uncertainty being acknowledged in biomedical text mining as an attribute of text mined interactions (events), it is significantly understudied as a means of providing a confidence measure for interactions in pathways or other biomedical models. In this work, we focus on improving identification of textual uncertainty for events and explore how it can be used as an additional measure of confidence for biomedical models.

**Results:**

We present a novel method for extracting uncertainty from the literature using a hybrid approach that combines rule induction and machine learning. Variations of this hybrid approach are then discussed, alongside their advantages and disadvantages. We use subjective logic theory to combine multiple uncertainty values extracted from different sources for the same interaction. Our approach achieves F-scores of 0.76 and 0.88 based on the BioNLP-ST and Genia-MK corpora, respectively, making considerable improvements over previously published work. Moreover, we evaluate our proposed system on pathways related to two different areas, namely leukemia and melanoma cancer research.

**Availability and implementation:**

The leukemia pathway model used is available in Pathway Studio while the Ras model is available via PathwayCommons. Online demonstration of the uncertainty extraction system is available for research purposes at http://argo.nactem.ac.uk/test. The related code is available on https://github.com/c-zrv/uncertainty_components.git. Details on the above are available in the Supplementary Material.

**Supplementary information:**

Supplementary data are available at *Bioinformatics* online.

## 1 Introduction

Advances in computational modelling support experimental simulations and facilitate biomedical pathway network analysis and construction. The use of pathways and protein interaction networks is becoming increasingly acknowledged and necessary (Pujol *et al.*, 2010), and there has been a surge in tools for visualization and processing of such networks (Pavlopoulos *et al.*, 2008). However, pathway curation (PC) and maintenance is still largely manual and time-consuming, partly because of the vast amount of literature that has to be reviewed.

**Fig. 1.**
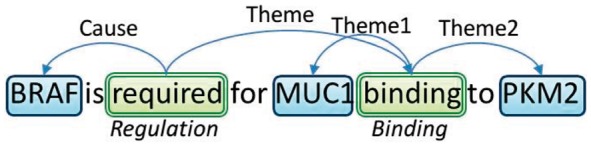
Event structures according to the *BioNLP* schema. Event triggers are enclosed in double-lined (green) boxes, while named entities (NEs) in single-lined (blue) ones. Arguments of events are represented by arrows above the words. We can observe that the *Regulation* event is a complex event, having the *Binding* event as its *Theme* argument

Text mining can aid PC (Hoffmann *et al.*, 2005; Oda *et al.*, 2008) and has already been used to link textual evidence from the literature (Ananiadou *et al.*, 2015; Miwa *et al.*, 2013). Current approaches focus on extracting bio-entities and interactions from papers referring to a pathway, either as supporting evidence (Miwa *et al.*, 2013; Vailaya *et al.*, 2005) or to reconstruct a specific pathway (Šarić *et al.*, 2005; Soliman *et al.*, 2016). Methods for linking evidence to interactions include extracting co-occuring entities, relationships between entities, or extracting more complicated interaction mentions (*n*-ary relations between entities) called *events* (Fig. 1). Since providing simple evidence for each interaction is far from sufficient, much work has focussed on providing measures of quality and confidence for each interaction. So far, such ‘scoring’ efforts have focussed on using entity co-occurrence statistics (Donaldson *et al.*, 2003; Szklarczyk *et al.*, 2011) or on employing experimental assays and methods in terms of biomedical confidence (Bader *et al.*, 2004; Schaefer *et al.*, 2012).

However, there has been little work on assessing the confidence of related information in terms of the certainty of a statement based on its textual context. Indeed, not all interactions mentioned in an article constitute facts or certain observations. They can be part of a hypothesis, a speculated outcome of an experiment, a case under investigation or a result attributed to an unclear external source (Medlock, 2008). Uncertainty of this type has been recognized and studied extensively for scientific documents, although mostly at the sentence level. Moreover, uncertainty and negation are acknowledged attributes of events and relations, annotated in the BioNLP (Kim *et al.*, 2009, 2011; Nédellec *et al.*, 2013) and CLEF (Mowery *et al.*, 2014) shared tasks, but never as a separate task. Indeed, there is little work focussing only on uncertainty of events and particularly linking it with interactions in pathways and interaction networks.

We go beyond existing efforts by extracting events with uncertainty values while relating them to existing pathway models. We propose the use of (un)certainty as an additional measure of confidence for interactions supported by evidence from literature. (Un)certainty-based confidence will help humans not only to quickly identify facts, but also to more rapidly synthesize hypotheses from highly uncertain interactions that are otherwise not intuitive or would be hard to identify in the literature.

We develop methods for (un)certainty identification of events and we provide a framework for consolidating (un)certainty values from several events to rank interactions accordingly. (Un)certainty is analysed from a textual point of view, as an attribute of each event in text, and consequently as an attribute of the corresponding interaction represented in a pathway.

We implement a hybrid framework that combines an automated rule induction approach with machine learning to discriminate between certain and uncertain interactions in text. As we show in Section 4.1, the combination of a Random Forest classifier with rule induction, which captures dependency patterns, boosts performance in terms of both recall and precision. We evaluate our work on two gold-standard corpora containing uncertain statements, which have been annotated by domain experts: GENIA-MK (Thompson *et al.*, 2011) and BioNLP Shared Task (BioNLP-ST) data Our hybrid approach outperforms previously reported performance, obtaining an F-score of 0.88 on GENIA-MK and 0.76 on the BioNLP-ST data. We extract supporting evidence for interactions contained in a pathway to determine the (un)certainty of each event. Subsequently, for those interactions with multiple evidence passages, we consolidate (un)certainty values from each event using subjective logic theory (Jøsang, 2001). This allows us to rank interactions proposed for a pathway model according to their associated (un)certainty, using a score for each interaction that takes into account the textual (un)certainty for all evidence. We present the evaluation carried out by domain experts/curators against two use-cases, thus confirming the validity of our approach.

## 2 Related work

Biomedical events (events for short) are centred around a trigger, i.e. a word or word sequence that denotes the occurrence of the event and the type of information expressed by it (referred to as the event type). An event has one or more arguments which are semantically linked to the trigger and contribute towards the event description. Arguments can be either named entities (NEs), or events themselves, in which case they are referred to as nested events, while the event that takes another event as its argument is considered a complex event. Arguments are categorized using semantic role labels that indicate the nature of their contribution to the event. The same entity can participate in different events, potentially assuming different roles in each event (Ananiadou *et al.*, 2015; Van Landeghem *et al.*, 2013). Figure 1 shows an example of event structures in biomedical literature.

The availability of a number of corpora annotated with events, such as Genia (Kim *et al.*, 2003), Multi-level Event Extraction (MLEE) (Pyysalo *et al.*, 2012), Gene Regulation Event Corpus (GREC) (Thompson *et al.*, 2009) and corpora used for the BioNLP-ST has supported the training of supervised models for event extraction. State-of-the-art performance in event extraction surpasses 0.55, reaching 0.76 in F-score for some event types (Björne and Tapio, 2015; Miwa and Ananiadou, 2015). This performance is high enough to obtain meaningful instances of events, rendering event extraction technology sufficiently mature to be used in a range of applications. Events are used to represent various types of bio-molecular interactions in scientific text, which can be mapped to pathway models (Miwa *et al.*, 2013; Björne *et al.*, 2010; Rzhetsky *et al.*, 2009).

**Fig. 2.**
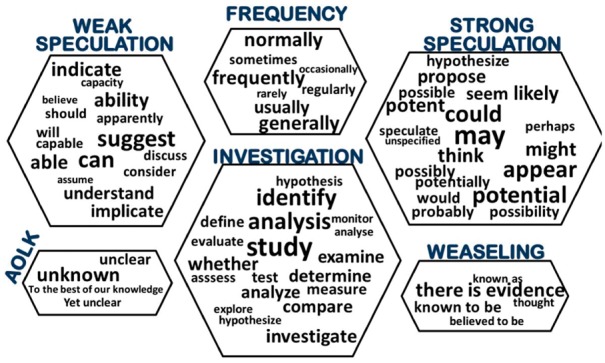
Uncertainty cues considered in the experiments grouped according to category (Strong/Weak speculation, frequency, Admission of lack of knowledge, Weaseling). Word clouds were generated based on BioNLP-ST and GENIA-MK

The same event can occur in different documents, but may be described as being more or less certain in each case, depending on context (words or phrases that modify the event without being part of it). As illustrated in Figure 2, the uncertainty of an event can be attributed to different constructs ranging from speculation and hedging to investigation or weaseling. Phenomena related to the expression of textual uncertainty have been studied at the sentence level using different terms, such as epistemic modality, speculation, factuality and hedging. Szarvas *et al.* (2012) propose a hierarchical categorization which distinguishes two main classes: hypothetical and epistemic uncertainty, while Medlock (2008) classify hedges as: extrapolated conclusions, relays of hedges from other work, limited knowledge, anaphoric hedging, questioning and hypothesis. Light *et al.* (2004) categorized uncertain statements as having high or low speculation. The BioScope corpus (Vincze *et al.*, 2008) comprises annotated biomedical sentences with speculation cues and their scope (Scope is defined as the whole sub-phrase affected by a speculation cue, as opposed to the event that is targeted by the cue).

Machine learning algorithms paired with dependency-based features have proven to be particularly effective in detecting speculation and scope both in the BioScope corpus and in the biomedical subtask of CoNLL 2010 that followed the cue-scope approach (Farkas *et al.*, 2010). Top performing systems in CoNLL 2010 achieved F-scores close to 0.86 using Conditional Random Fields (Tang *et al.*, 2010). More recent approaches focus on optimizing scope detection, using either dependency-based methods (Zhou *et al.*, 2015; Zou *et al.*, 2013), or a combination of rule-based methods and machine learning algorithms (Velldal *et al.*, 2012).

In contrast to the cue-scope approach, the BioNLP-ST and the GENIA-MK corpus introduce the idea of (un)certainty for a specific event rather than a sub-phrase of a sentence (see Section 3.1). When compared with performance reported for the cue-scope approach tasks, the performance for the speculation identification subtask of the BioNLP-ST was particularly low; the best result achieved on the Epigenetics (EPI) task was an F-score of 0.38 (Björne and Salakoski, 2011). However, Stenetorp *et al.* (2012b) used a combination of rule-based heuristics and a Support Vector Machine classifier using cue-scope and event-based features, to obtain 0.52 F-score for the EPI task, and to improve the results of the systems in the BioNLP 2011 by at least 4%. On GENIA-MK, the best performance so far comes from Kilicoglu *et al.* (2015), who obtained F-scores of 0.67 and 0.68, respectively for the L1 and L2 classes of GENIA-MK annotations (see Section 3.1). Currently there is no standard, commonly accepted categorization of uncertainty and epistemic modality in text (Rubinstein *et al.*, 2013). Thus, there are different interpretations of these concepts across different corpora and experiments, leading to significant disagreement in terms of how uncertainty is annotated in text.

Aiming for a wide coverage of diverse uncertainty expressions, we chose to consider ‘uncertainty’ as corresponding to all cases described in previous work as indications of speculation, hedging or epistemic modality. The scope of our definition of uncertainty is illustrated by the general categories represented in Figure 2, each accompanied by example uncertainty cues. The categories are further elaborated in the Supplementary Material, Section 2, in which we also provide details of the coverage of such categories in different corpora. Although not all of the uncertainty cues are equally strong in terms of hedging an event, in this work we consider all events that are modified by an expression belonging to one of the categories shown in Figure 2 to be classed as uniformly uncertain, leading to a binary classification problem definition for uncertainty.

Various studies have exploited text mining to support biomedical network construction and PC (Hoffmann *et al.*, 2005; Shatkay and Feldman, 2003). Inferring a biomedical network from textual resources is a complicated task, typically requiring the combination of several text mining processes. Czarnecki and Shepherd (2014) analyse the process of constructing protein–protein interaction (PPI) networks and specify the necessary components for a text mining pipeline to achieve this. Along the same lines, Subramani *et al.* (2015) extracts potential protein relations from text, and uses databases to validate them, map them to pathways and visualize the result. However, this process seems dependent on the database information without additional confidence measures. Malhotra *et al.* (2013) extracted hypothesis statements (overlapping with uncertain statements) from text to build hypothetical stage-specific disease networks. Although they describe different degrees of uncertainty they do not use this information to rank interactions in the networks. Soliman *et al.* (2016) also present the construction of an interaction network from text mining, but they use reference interaction databases in order to classify the extracted relations in terms of validity and knowledge novelty. Finally, although not related to networks, Jilani *et al.* (2008) use speculation markers to classify—in terms of confidence—statements from biomedical papers relating to the apolipoprotein E gene.

Oda *et al.* (2008) links events to pathways while highlighting the difficulties to map and integrate multiple textual fragments to the same pathway node. PathText2 (Miwa *et al.*, 2013) uses event extraction and links evidence from the literature with pathway models to return ranked evidence pertaining to the interactions described in the model. STRING database (Szklarczyk *et al.*, 2011) scores interaction networks based on co-occurrence statistics of the participating entities (along with experimental assay scoring). Donaldson *et al.* (2003) also proposes a text mining approach to support PPI curation and provides a confidence score based on the co-occurrence of protein mentions. However, in that work, textual uncertainty expressed in the evidence passages was not considered, and there was no distinction between certain and uncertain statements.

These past efforts contributed to the automation of PC and enhancement of biomedical networks, and illustrate potential uses of textual uncertainty for biomedical purposes. However, the scope of each application is limited and textual uncertainty is rarely considered when linking evidence to pathways. In addition, in efforts to extract uncertainty from biomedical corpora, there has often been a lack of experimental evaluation or validation of an application by domain experts.

## 3 Materials and methods

We present our methods for assessing and ranking pathway interactions based on (un)certainty. In Section 3.1, we describe the datasets and models that were used. Section 3.2 details our hybrid approach for identification of uncertain events, while Section 3.3 describes the method used to combine multiple events mapping to the same interaction in one consolidated value. The text-mining workflows based on our methods are available and described in the Supplementary Material, Section 1.

### 3.1 Datasets, models and evaluation

To identify the uncertainty of events in text, we leverage the event-annotated corpora developed for the BioNLP-ST, and the GENIA-MK corpus, both for training and testing purposes.

GENIA-MK consists of 1000 biomedical abstracts annotated with events. Each event has high-level information (or meta-knowledge) annotations (Thompson *et al.*, 2011), including separate annotations for Certainty Level (CL) and Knowledge Type (KT). These are both mapped to binary uncertainty values for our evaluation. In terms of CL, there are 3 different classes: L1 (‘considerably speculative’), L2 (‘somewhat speculative’) and L3 (‘non-speculative’). KT classes include Investigation, Analysis, Observation and General. We consider the cases annotated as L1, L2 or Investigation to fall under our definition of uncertainty, so we use them to generate instances of uncertain events for training and testing. Based on this definition, 8.1% of the 36 858 events are classified as uncertain.

In BioNLP-ST 2009, 2011 and 2013, a wide range of subtasks included events that were annotated with binary speculation values. The tasks with speculation attributes are: Cancer Genetics, PC, EPI, Infectious Diseases (IDs) and GENIA (GE). All events annotated as speculated are considered as uncertain based on our uncertainty definition. For our experiments, we took the union of the datasets provided by the above BioNLP tasks, which we collectively refer to in this work as the BioNLP-ST corpus. For both GENIA-MK and BioNLP-ST, we evaluate the performance of our methods using 10-fold cross validation, calculating precision, recall and F-score in each case.

In order to evaluate our approach to ranking pathway interactions according to the (un)certainty of related events in text, we evaluated our results based on two pathway use-cases

1. A manually curated pathway model, *B-cell Acute Lymphoblastic Leukemia Overview* (henceforth referred to as the Leukemia Model), from Pathway Studio disease collections (https://mammal.pathwaystudio.com/#nav-5). This model includes 103 biomedical entities and 179 interactions, with each interaction accompanied by related evidence (small passages) from published papers, manually selected by curators. We automatically extracted events from those passages, calculated their (un)certainty values and then ranked each interaction based on these values.
2. A two-hop neighbourhood network of the Ras gene generated for the Big Mechanism project (Cohen, 2015) and a collection of full-text papers extracted from PubMed, focusing on Melanoma (henceforth referred to as the Ras-melanoma Model). The model was generated by querying the Pathway Commons API (//www.pathwaycommons.org/pc2/graph?source=P01112&source=P01116&source=P01111&kind=neighborhood). The papers were annotated with events using EventMine (Miwa *et al.*, 2012), and sentences that contained events mapping to the Ras-melanoma model were grouped and mapped to their corresponding interaction in the network. Uncertainty identification methods were then applied to the linked sentences, to classify mapped events as certain/uncertain and to score the related interactions in terms of textual (un)certainty.

The results were then presented to domain experts for evaluation, as described in Section 4.2.

### 3.2 A hybrid approach for (un)certainty identification

Our approach to textual (un)certainty identification is based on a combination of two components: (i) machine learning classification and (ii) rule induction. Both components perform binary classification of an event, where the set of possible classes is {certain, uncertain}. Comparing different combinations of the two components, we found that when the induced rules are used as features, the machine learning component obtained the best performance. Details of the implementation are described in the following sections, and the results are provided in Section 4.

#### 3.2.1 Rule induction

**Fig. 3.**
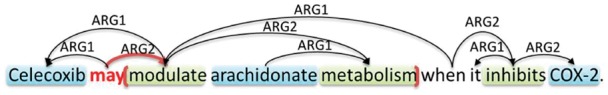
Relation between the influence of uncertainty cues and syntactic dependencies. Dependencies are marked with arrows above text, while the scope of the uncertainty cue *may* is marked with the red squared brackets (Color version of this figure is available at *Bioinformatics* online.)

The existence of an uncertainty cue such as *possibly* or *suggest* in a sentence will not necessarily render any event in the same sentence uncertain, as illustrated in Figure 3, where the only uncertain event is the one that has the word *modulate* as a trigger. The event with *metabolism* as a trigger, while syntactically within the scope of *may* (indicated by red squared brackets), is not within the scope of the uncertainty. Similarly, *inhibition of COX-2* is not affected by the presence of *may*. The results of dependency parsing (marked with arrows above the sentence), can help to identify which event triggers are directly dependent on the uncertainty cue. Thus, dependency parsing can provide useful insights into the way a cue affects the trigger of each event in a sentence. In many cases, event (un)certainty can be determined from the dependency path between a cue and a trigger. Indeed, compared with the other event triggers in the sentence of Figure 3, the dependency path between *may* and *modulate* is the shortest one, as there is a direct dependency identified between the two words. Our rule pattern induction looks for generic rule patterns that can capture dependency relationships between (un)certainty cues and trigger words, which extend to multi-hop dependencies.

To extract dependency graphs over a tokenized sentence, we use the Enju dependency parser (Matsuzaki and Tsujii, 2008). We then extract dependencies between two tokens as directed edges from the source token *T*_s_ (dependency head) to the target token *T*_t_. Hence, we can define a dependency function whose output is the type of dependency that takes values from a closed set of labels provided by Enju (ARG1: subject of a verb, a target of modification by modifiers etc. ARG2: object of verbs, prepositions, etc. ARG3/ARG4: objects and complements of verbs etc. MOD: participial constructions etc. Denotes a clause modified by another clause, if the subordinate clause has an ARG1).

(1) $$\text{dep}(T_{S},T_{T})=d,d\in [ARG1,ARG2,ARG3,MOD,\emptyset ]$$

The output may include the null value in the case where there is no dependency between the two tokens. Based on the definition of Equation (1) we can also define ‘dependency chains’ as sequences of consecutive dependency edges that create a directed path between a source *T*_S_ and a target *T*_T_ token (see Equation 2). If a sentence contains a non-empty chain (*T*_S_, *T*_T_), where *T*_S_ corresponds to an (un)certainty cue and *T*_T_ to an event trigger, it is considered a valid pattern and rule candidate, formulated as Equation (3).

(2) $$\text{chain}(T_{S},T_{T})=\{\begin{array}{l} \text{dep}(T_{S},T_{T}),\text{if}\;\text{dep}(T_{S},T_{T})\neq \emptyset \\ \text{chain}(T_{S},w)\cdot w\cdot \text{chain}(w,T_{T}),\\ \;if\exists w:\text{chain}(T_{S},w)\neq \emptyset ,\text{chain}(w,T_{E})\neq \emptyset \\ \emptyset ,\text{else} \end{array}$$

(3) $${\text{Rule}}_{i}=T_{S}\cdot \text{chain}(T_{S},T_{T})$$

We present in the Supplementary Material, Section 3.2.1 an example of step-by-step application of Equations (1)–(3) on a dependency-parsed sentence in order to derive a rule, and the application of the same rule to a new sentence.

When extracting rules from unannotated data, a list of potential uncertainty cues is necessary to guide the rule pattern extraction. The availability of a corpus with events annotated with uncertainty cues allows us to extract all chains around an uncertain event trigger as potential rules. One or more measures of informativeness can be used to filter the potential rules and retain only the most meaningful ones. We experimented with different measures and decided to use *Interest* (Brin *et al.*, 1997) as it was better at distinguishing patterns containing uncertainty cues from the ones containing irrelevant ones (see Supplementary Material, Section 3.2.2.1). In both cases, the size of the extracted rule-set can be further reduced by applying task-specific constraints to retain only the most meaningful rules and downsize the search space. Constraints were applied to the extraction of (un)certain events. Specifically: (i) for each (un)certainty cue, we limit the rule generation to the event most directly affected by that cue. No token *w* in a chain should belong to a trigger of another event. (ii) We constrain the maximum length (*n*) of a *chain*() function to *n* < 3. See the Supplementary Material, Section 3.2.2.2 for details on pattern coverage.

Although the automated rule extraction system can accurately extract dependency patterns, it may fail to account for other features within a sentence. Our hybrid approach complements the rule-based component with additional machine learning features. We applied a Random Forest classifier (Liaw and Wiener, 2002) using a set of linguistic features covering semantic, lexical and syntactic aspects (Supplementary Material, Section 3.1).

### 3.3 (Un)certainty-based confidence measure for model interactions

Using the methods described in Section 3.2, any interaction can be classified in terms of its (un)certainty. The literature often includes multiple references to the same interaction, whose (un)certainty levels can vary. To determine an overall confidence measure for a single interaction, we need to consolidate the binary (un)certainty values extracted from multiple evidence passages.

We chose to use subjective logic theory (Jøsang, 2001) to obtain a consolidated score for each interaction, derived from the binary values of the text mined events. Each evidence sentence that contains an event *e_x_* mapping to a pathway interaction *i_x_*, can be considered as the subjective opinion of the author for the interaction *i_x_*. According to Jøsang, if *x* is a proposition, a binomial opinion about the truth of *x* is the ordered quadruple $\omega_{x}=(b,d,u,\alpha )$, where:

- *b*: belief is the belief that the specified proposition is true.
- *d*: disbelief is the belief that the specified proposition is false.
- *u*: uncertainty is the amount of uncommitted belief.
- *α*: base rate is the *a priori* probability in the absence of evidence.

and the condition in Equation (4) must always be satisfied. Then, the probability expectation value (E) of an opinion is defined in Equation (5).

(4) b+d+u=1,∀b,d,u,α∈[0,1]

(5) E=b+α·u

Assuming we have several different opinion sources (authors) referring to the same proposition (interaction) with different levels of certainty, we can fuse their opinions based on subjective logic. Different fusion formulas have been suggested (Jøsang *et al.*, 2006), but we choose to follow the cumulative fusion that is suited for independent opinions and considers the amount of sources as well. Since we want to consider fusion of multiple sources we use the formula suggested by (Jøsang *et al.*, 2017), in order to combine belief $b_{X}^{C}(x)$ and uncertainty $u_{X}^{C}$ from each source (C∈ℂ) to the fused belief $b_{\bar{X}}^{⋄(\mathbb{C})}$ and uncertainty $u_{\bar{X}}^{⋄(\mathbb{C})}$ that will allow us to calculate the overall probability expectation value $E=b_{\bar{X}}^{⋄(\mathbb{C})}+\alpha \cdot u_{\bar{X}}^{⋄(\mathbb{C})}$. According to that formula in cases where there is at least one uncertain opinion ($\exists u_{X}^{C}\neq 0$) we have:

(6) $$\{\begin{array}{c} b_{\bar{X}}^{⋄(\mathbb{C})}=\frac{\sum_{C\in \mathbb{C}}(b_{X}^{C}(x)\cdot \prod_{C_{j}\neq C}u_{X}^{C_{j}})}{\sum_{C\in \mathbb{C}}\prod_{C_{j}\neq C}u_{X}^{C_{j}}-(N-1)\cdot \prod_{C\in \mathbb{C}}u_{X}^{C}}\\ u_{\bar{X}}^{⋄(\mathbb{C})}=\frac{\prod_{C\in \mathbb{C}}u_{X}^{C}}{\sum_{C\in \mathbb{C}}\prod_{C_{j}\neq C}u_{X}^{C_{j}}-(N-1)\cdot \prod_{C\in \mathbb{C}}u_{X}^{C}} \end{array}$$

In this way, subjective logic allows us to define *b* and *u* as the certainty and uncertainty of an interaction respectively. Moreover, we can model in a straightforward way the cases of negated events (where the event is contradicted but with no indication of uncertainty) as disbelief *d*. To identify negated events, we can use methods similar to uncertainty as described in Nawaz *et al.* (2013).

We also choose subjective logic because it accounts for uncertain opinions while maintaining compatibility with standard logic and probability calculus. We limit our work to explore the potential of fusing statements that are considered independent to the rest. However, in Jøsang *et al.* (2006), there is substantial theory on combining dependent or partly dependent opinions as well as on propagation of opinions within networks and attributing different certainty to different sources, that we intend to study in the future.

## 4 Results

### 4.1 Comparative evaluation

We evaluated our approach on the GENIA-MK corpus, and the BioNLP-ST corpus as described in Section 3.1. Rule selection and feature extraction was guided by a pre-selected list of 60 uncertainty cues that was compiled based on the GENIA-MK and BioNLP-ST corpora, as well as related publications (Malhotra *et al.*, 2013; Rubin, 2007). In Table 1, we compare the performance of our combined hybrid system against each of the components when used individually.

**Table 1.** Comparative evaluation on GENIA-MK and BioNLP-ST corpora

Corpus	System	Precision	Recall	F-score
GENIA-MK	ML only	0.79	0.67	0.72
	Rules only	0.81	0.52	0.63
	ML + Rules	0.76	0.77	0.77
	ML + Rule features	0.94	0.83	**0.88**
BioNLP-ST	ML only	0.82	0.64	0.73
	Rules only	0.42	1.0	0.59
	ML + Rules	0.35	0.77	0.48
	ML + Rule features	0.87	0.68	**0.76**

Values in bold indicate best performance obtained for each corpus.

The best results are obtained by using the induced rule patterns as features for the Random Forest classifier. It is important to note that the performance on the GENIA-MK corpus is consistently higher for all different systems. We attribute this to the fact that the BioNLP-ST corpus consists of different corpora with differences in their annotation procedure. As a point of comparison, the best performance on BioNLP-ST is reported by Stenetorp *et al.* (2012b) who, for BioNLP 2011, obtained an F-score of 0.52 for the EPI track, 0.40 for the GE track and 0.37 for the ID track. The rule-based features, that can account for rule patterns that span to 2-hop dependency relations (prior work, such as Kilicoglu *et al.*, 2015; Xu *et al.*, 2015 focusses on one-hop dependencies), contributed considerably to the improvement of the performance.

The specially selected cue-list also contributes to boosting the performance, as it plays a crucial role in in the rule and feature selection. We carried out two additional experiments, to determine the generalization and portability of our method to other domains. These are presented in Table 2 and demonstrate the extent to which the selection of the initial cue list affects performance. First, we want to assess the portability of our method, and the domain specificity of our definition of (un)certainty. So we replace our list with cue lists from the general domain (Automatic Content Extraction (ACE) corpus; Thompson *et al.*, 2016). Although intuitively we assumed that phrases expressing (un)certainty are domain-independent, it turned out that the range of expressions in the general/newswire domain is wider and the cues are more complicated (often multiword, colloquial expressions) leading to considerably decreased performance for both corpora.

**Table 2.** Comparative evaluation on GENIA-MK and BioNLP-ST corpora using different approaches for rule extraction and cue identification

Corpus	System	Precision	Recall	F-score
GENIA-MK	Bio cues	0.94	0.83	**0.88**
	ACE cues	0.82	0.86	0.84
	No cues	0.93	0.67	0.78
BioNLP-ST	Bio cues	0.87	0.68	**0.76**
	ACE cues	0.61	0.53	0.58
	No cues	0.86	0.66	0.74

Second, we chose not to constrain the rule pattern generation with pre-selected cues. Instead, we extracted all potential two-hop length rule patterns around uncertain events, obtaining a pattern-set that included both patterns indicating uncertainty and meaningless ones (for our task). Patterns were then sorted according to *Interest* measure (Brin *et al.*, 1997), in order to maintain only the ones with the highest score and that contained a higher percentage of uncertainty cues (see Supplementary Material, Section 3.2.2.1). Hence the cue list was automatically compiled by those patterns (removing stopwords). Although the performance dropped on both corpora, it still produced reasonable results, and the precision remained high. Indeed, the compromise in this case was mainly in terms of recall, since as shown in the Supplementary Material, some of the correct rule patterns are lost during filtering. However, in the case of the BioNLP-ST corpus, which is substantially larger than GENIA-MK, the drop in recall is considerably smaller. This result is promising, and paves the way for further experiments towards semi-supervised (un)certainty identification.

### 4.2 Application to pathway models

Having validated our methods for uncertain event identification on gold standard corpora, we applied them, together with our adaptation of subjective logic theory described in Section 3.3, to interactions described in pathway networks. We used the Leukemia and Ras-melanoma models as described in Section 3.1. For both use-cases we firstly applied EventMine to the evidence passages to identify and map the events in each passage to the model interactions. We then applied our (un)certainty identification system to the results of EventMine using Equation (6) to calculate the fused (un)certainty score for each interaction. The automatically annotated events and interactions were then evaluated by domain experts using the brat annotation tool (Stenetorp *et al.*, 2012a) which presented each interaction with its related evidence. The evaluation interface can be accessed on brat (http://nactem10.mib.man.ac.uk/bratv1.3/#/Pathway_Annotations/) and the annotation guidelines are available online (https://tinyurl.com/y7776ztl).

#### 4.2.1 The leukemia use-case

For most interactions in the Leukemia pathway model, there is at least one evidence passage provided as a reference, but the number of evidence passages can surpass 100 for some interactions. As stated in the Pathway Studio manual (http://tinyurl.com/gsywlar), the only confidence measure provided for the interaction simply reflects the number of associated evidence passages and ranges from 0 (none) to 3 (≥3 related publications). Such a measure is not always indicative of the confidence attributed to an interaction, since an evidence passage may contain uncertainty which should be taken into account. We therefore propose the application of an (un)certainty-based confidence measure based on Equations (4)–(6), that reflects the (un)certainty found in the textual evidence.

Seven domain experts were asked to evaluate a total of 72 interactions, each of which was accompanied with evidence passages. Overall, 260 evidence passages (with from 1 to 20 passages for each interaction) were evaluated, of which 12% were flagged as uncertain by our system. Each evaluator was presented with the decision of our system for each evidence sentence (event) separately, as well as the overall decision for each interaction, and was asked to state their agreement/disagreement for each sentence. In terms of the interactions, we consider as correct, only the cases where the annotators agreed with all the sentence annotations. The results are presented in Table 3.

**Table 3.** Recall, precision, F-score and accuracy (on sentence and interaction level) of system annotations according to evaluation by seven annotators (A1–A7)

	A1	A2	A3	A4	A5	A6	A7	MAvg	SD
Precision	0.93	0.83	0.86	1	1	0.74	0.88	**0.89**	0.09
Recall	0.86	0.57	0.63	0.78	0.79	0.69	0.59	**0.70**	0.11
F-score	0.89	0.68	0.73	0.86	0.89	0.71	0.71	**0.78**	0.09
Acc. per int.	0.93	0.93	0.85	0.91	0.9	0.79	0.81	**0.87**	0.06
Acc. per sent.	0.98	0.98	0.92	0.97	0.97	0.94	0.93	**0.96**	0.03

*Note*: Mean average (M Avg) and Standard deviation (SD) measures also provided.

We used a set of 10 validation sentences among the 260, in order to verify the consistency of the evaluators (validation sentences were pre-selected sentences considered to have a very clear certain/uncertain value, but were purposely assigned erroneous labels in order to verify that annotators were attentive and consistent during the task). We then calculated the inter-annotator agreement (IAA) over the whole set of 260 sentences in pairs (Supplementary Material, Section 4.1.1) that gave a mean average Kappa value of 0.65. The IAA agreement levels range from moderate (0.53) to very good (0.82), showing that the perception of (un)certainty can vary among different users. It is also noticeable that, in cases of annotators disagreeing with the output of the system, the disagreement results from humans perceiving even more sentences than the ones selected by the model as uncertain, thus leading to low recall.

Nevertheless, the overall performance of our system confirms the usefulness of our (un)certainty-based confidence measure, which can complement and enhance the simpler measure currently provided by Pathway Studio. The results provide a solid base for further experiments presented in Section 4.2.2 and the Supplementary Material, Section 4.1.2, where we present a more extensive quantitative evaluation both on sentence and interaction level.

#### 4.2.2 The Ras-melanoma use-case

Since the Ras gene plays a central role in many cancer cases, the Ras-melanoma model could be an important resource for research if supported by sufficient evidence from the literature. Indeed, as it contains more than 100 interactions and genes, it is feasible to identify a large number of related sentences and events in the literature, particularly since the Ras gene and related interaction play a key role in cancer-related research. Due to the large amount of related statements, a method for ranking interactions in terms of (un)certainty of the evidence can facilitate faster filtering of information. In this experiment, we focus on the scoring and quantification of (un)certainty and assess evidence on a 1–5 scale.

We asked two experts to annotate a total of 100 interactions, each with 1–10 associated evidence passages, amounting to 392 passages in total. They were asked to assess the certainty of the event in each evidence sentence on a scale of 1–5, where 1 corresponds to ‘most uncertain’ and 5 to ‘most certain’. Subsequently, the annotators were asked to score each interaction in the pathway, based only on the associated evidence.

**Fig. 4.**
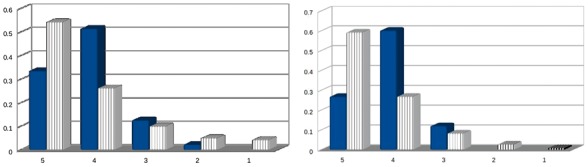
Distribution of scores for (un)certainty between annot. 1 (solid colored (blue) bars) and annot. 2 (vertically stripped white bars) (Color version of this figure is available at *Bioinformatics* online.)

The distribution of scores is presented in Figure 4. It is worth noting that while there is no total agreement, both annotators annotated the majority of sentences with high certainty (≥4). However, it is clear that the perception of (un)certainty varies, since, for example, the scoring of annotator 1 is shifted towards higher certainty values. The overall agreement at the sentence level was 43%, but only in 8% of the sentence annotations was the difference in the assigned score greater than 1. For interactions, the overall agreement was 45%, and only for 8% of cases where the disagreement was >1 point on the scoring scale.

**Fig. 5.**
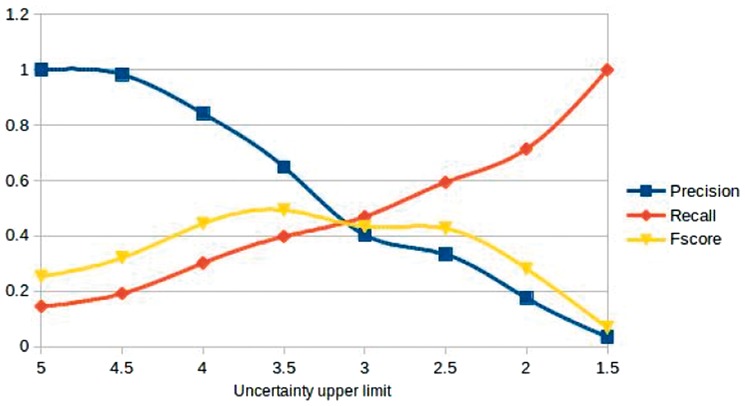
Performance in terms of precision, recall and F-score, depending on the selection of the mean average score as the upper limit of uncertainty (i.e. the value below which all scored events must be considered uncertain)

In this non-binary evaluation, mapping the results of the sentence annotation to our binary methods was more complicated. In Figure 5, we present precision, recall and F-score plotted against different upper limits for uncertainty, showing the trade-off between precision and recall in each case. In the extreme values, our system performs well, i.e., there was no case where it annotated as uncertain a sentence where the mean average score was 5. Similarly, in the few cases where the mean average score was 1.5, our system picked up the uncertainty of the event. As expected, for stricter uncertainty upper limits, recall rises, but precision drops, while the best performance (0.50 F-score) is obtained for the limit set in the mean average of 3.5. Such results indicate that while the binary method performs consistently, looking into a finer grained quantification of (un)certainty would be a worthwhile goal for future work, to more closely mirror the perception of users.

Focussing on the scoring of the interaction itself, we evaluate the subjective logic fusion as follows. Firstly, we consider the results of our system under the following assumptions: (i) *α* is set to 0.5 for all cases, (ii) an event *e*1 that maps to interaction *x* and is identified as uncertain, constitutes an opinion with $b_{x}^{e1}=0.5,u_{x}^{e1}=0.5$ and (3) an event *e*2 that maps to interaction *x* and is identified as certain has $b_{x}^{e2}=1,u_{x}^{e2}=0$. Also we project the 1–5 scoring to a (0,1) scale by dividing by 5. Thus, we can use Equations (5) and (6) to calculate the score of the interaction *x* and compare it to the scores given by the annotators. We calculate the absolute difference between the score of our system and the mean average score given by the annotators. The results are shown in Table 4 where we also present the mean average absolute difference between the score given to the interaction by each annotator and the score calculated with Equation (6) using the scores given by the same annotator for each event mapped to the interaction.

**Table 4.** Performance results for the interaction scoring on the Ras-melanoma model

	Ann1	Ann2	Mean Avg (1 and 2)	System prediction
Mean Avg Diff	0.06	0.09	0.07	0.13
SD	0.06	0.12	0.09	0.11

We can observe that the score predictions when using the scores given to the events by the annotators are very close to the actual scores attributed by them. The score given by the system deviates slightly more, but this was to be expected, since our system uses binary classification of (un)certainty. Hence, subjective logic provides a good approximation of the score and way users assess (un)certainty based on a series of statements by different authors. We consider this to be an encouraging step towards combining certainty from different sources. This is especially so, because it will accommodate future approaches that consider further (un)certainty parameters to better approximate user scores and to take into account phenomena such as the same event being mentioned multiple times in one document, or uncertainty expressions being used consistently as part of a writing style rather than a way to convey hesitation on a statement.

## 5 Conclusion

In this article, we have focussed on the analysis and interpretation of textual (un)certainty in relation to events and demonstrated how this can support scoring of pathway interactions. We have proposed (un)certainty scoring as a more expressive confidence measure, to be used as an alternative or to complement simpler frequency-based evidence measures.

We used a broad definition of textual uncertainty and developed a hybrid framework for (un)certainty identification, which combines rules with machine learning. Our proposed methods identify an (un)certainty value for each event mentioned in a sentence separately, instead of the more commonly-used sentence-level (un)certainty identification. We then introduced an approach to consolidate uncertainty values from different papers into a single score, in order to directly account for the impact of textual uncertainty directly on the interaction of interest. This scoring can help to isolate the more controversial interactions from the ones for which there is wider agreement. This is an important step towards decreasing manual curation effort, since users can view the (un)certainty values of interactions, identify the interactions of interest and then selectively read through the publications related to the provided evidence.

It is important to note that the success of the proposed confidence measure requires robust performance of the (un)certainty identification method for individual events. For this reason, we have demonstrated that our hybrid methods, and the incorporation of rules that account for multi-hop dependencies, considerably outperform other published work based on similar gold-standard corpora. We have presented different approaches for rule extraction, and discuss the trade-offs between them. Implementation of the related components on a text-mining platform facilitates the incorporation of our system in different workflows based on the task at hand. Accordingly, the demonstration workflow presented in the Supplementary Material, Section 1.1, can be applied to identify new evidence from recent papers pertaining to the interactions of a model, thus aiding curators to keep the model up-to-date.

Evaluation on the pathway models shows the applicability of our methods on unseen data and verifies that use of subjective logic provides a confidence score that is a good approximation of scores attributed by experts. However, it is clear that broadening the definition of uncertainty and applying a finer-grained classification of uncertain statements will be an important future step to better approach the perception of users about uncertainty. To better approach the perception of (un)certainty by readers, we also intend to focus our future work on expanding the use of subjective logic to account for phenomena such as propagation of (un)certainty via citations and multiple or dependent events mentioned by the same author etc. We also want to study varying trust/certainty in opinions of different authors that would allow us to account for cases where authors consistently use uncertainty expressions due to writing style versus authors who tend to write in a more assertive style.

All the above could further boost the performance of our presented method which, by detecting (un)certainty from text, can support PC based on big textual collections.

## Supplementary Material

Supplementary Data
