# Supplementary material for: Using uncertainty to link and rank evidence from biomedical literature for model curation
Source: Bioinformatics. 2017 Jul 24;33(23):3784–92. doi: 10.1093/bioinformatics/btx466 (PMC5860317; doi:10.1093/bioinformatics/btx466)
Supplement: Supplementary Data [file btx466_supplementary_material_revised.pdf]

Supplementary Material

1 Demonstration of uncertainty related workflows

In this section we present the details of the implemented workflows that are publicly available to demonstrate the current work. Both workflows are available on Argo platform (test server), that can be accessed here: `http://argo.nactem.ac.uk/test`.

For most of the work described in the main manuscript to be carried out, significant processing of the plain text documents is involved. Indeed building an end-to-end system that is able to parse full-text papers, relate them to interactions described in a pathway model, and rank those interactions according to the (un)certainty of the interaction mentions in the related evidence, is a multifaceted task consisting of several natural language processing (NLP) subtasks, ranging from the annotation of sentences with named entities, events and uncertainty to the matching of events to a pathway interaction. To combine the different NLP tools in a pipelined manner, we used the Argo platform Rak *et al.* (2012). Argo is a Web-based, graphical workbench that facilitates the construction and execution of modular text mining workflows. Underpinning it is a library of diverse elementary NLP components implemented according to the Apache UIMA OASIS standard[1], each of which performs a specific task.

For best results, please access the platform and workflows only on Firefox web browser.

A constantly updated version of this first section, titled *Demonstration of uncertainty related workflows* is available at `https://docs.google.com/document/d/1b0YBllgic-f750_t63nyjMQLsOVHkB8OImrQi1Wh5bc/edit?usp=sharing` where it is maintained and updated on a regular basis, to account for changes in the argo platform, new user requests, etc.

1.1 Access to necessary resources

All the related files are available on a NaCTeM server, *nactem10.mib.man.ac.uk*. An account has been created that can be accessed over SSH File Transfer Protocol (SFTP) connections using the following credentials:

- Username: workflow_tester
- Password: ul+raRiver47
- Home directory: /data/sftp/workflow_tester

Note that users have write access only under the uploads folder. By default, the following data is uploaded on the folder:

1. **leukemia_files**: Set of passages related as evidence to the interactions described in *B-cell Acute Lymphoblastic Leukemia Overview* pathway of Pathway Studio
2. **ras_melanoma_files**: Set of sentences extracted from full-text PubMed papers focussing on Melanoma disease and mapped to the nodes of the Ras 2-hop neighborhood network.
3. **leukemia.csv**: File containing the extracted information from the *B-cell Acute Lymphoblastic Leukemia Overview* pathway of Pathway Studio
4. **Genia-MK**: The Genia-MK corpus files in standoff annotation
5. **All_BioNLP**: The merging of the BioNLP-ST corpora in standoff annotation
6. **ras_models**: Directory that contains JSON snaphots of the normalised Ras model that was assembled the Big Mechanism project and was used for our Ras-Melanoma use case described in the main manuscript, Section 4.2.2. It contains:

[1] https://uima.apache.org

- **Ras.json**: Normalised JSON version of the 2-hop neighborhood of the Ras gene. The model was originally generated in BioPAX (owl) format, by quering the Pathway Commons API. The query used for the network generation is: `http://www.pathwaycommons.org/pc2/graph?source=P01112&source=P01116&source=P01111&kind=neighborhood`
- **Ras-melanoma.json**: Ras.json model, enhanced with sentences extracted from full-text PubMed papers focussing on Melanoma disease and mapped to the nodes of the original model. In this version, each interaction is accompanied with related evidence sentences (if any) along with uncertainty values for each event/sentence and fpr the interaction as a whole.

7. **model_resources**: Contains pre-trained models to be used in the workflow.
    - **Negation.model**: model trained on GENIA-MK to identify negated events. Needed for *WEKA Classifier (negation)* component.
    - **protein.model**: model for Protein named entity recognition. Needed for *NERsuite Tagger* component.
    - **genia.all.model**: model trained on GENIA-MK to identify uncertain events. Needed for *WEKA Classifier (uncertainty)* component. It is trained using the preselected biomedical uncertainty cue list, which should be used with the *Rule Feature Generator* component in the same workflow. It also requires the *Uncertainty Feature Generator* component to be used before the classifier.
    - **bio.all.model**: model trained on BioNLP-ST to identify uncertain events. Needed for *WEKA Classifier (uncertainty)* component. It is trained using the preselected biomedical uncertainty cue list, which should be used with the *Rule Feature Generator* component in the same workflow. It also requires the *Uncertainty Feature Generator* component to be used before the classifier.
    - **combined.all.model**: model trained on the combination of GENIA-MK and BioNLP-ST to identify uncertain events. Needed for *WEKA Classifier (uncertainty)* component. It is trained using the preselected biomedical uncertainty cue list, which should be used with the *Rule Feature Generator* component in the same workflow. It also requires the *Uncertainty Feature Generator* component to be used before the classifier.
    - **genia.all.ace.model**: model trained on GENIA-MK to identify uncertain events. Needed for *WEKA Classifier (uncertainty)* component. It is trained using the ACE derived (Thompson *et al.*, 2016) cue list, which should be used with both the *Rule Feature Generator ACE* and *Uncertainty Feature Generator ACE* components in the same workflow.
    - **bio.all.ace.model**: model trained on BioNLP-ST to identify uncertain events. Needed for *WEKA Classifier (uncertainty)* component. It is trained using the ACE derived (Thompson *et al.*, 2016) uncertainty cue list, which should be used with both the *Rule Feature Generator ACE* and *Uncertainty Feature Generator ACE* components in the same workflow.
    - **combined.all.ace.model**: model trained on the combination of GENIA-MK and BioNLP-ST to identify uncertain events. Needed for *WEKA Classifier (uncertainty)* component. It is trained using the ACE derived (Thompson *et al.*, 2016) uncertainty cue list, which should be used with both the *Rule Feature Generator ACE* and *Uncertainty Feature Generator ACE* components in the same workflow.
    - **genia.all.nc.model**: model trained on GENIA-MK to identify uncertain events. Needed for *WEKA Classifier (uncertainty)* component. It is trained using a list of rules automatically extracted from GENIA-MK using the lift formula to filter the meaningful rules (see Eq 3). The resulting uncertainty cue list, can be found in the *rule_resources* directory and should be used with both the *Rule Feature Generator Broad* and *Uncertainty Feature Generator Broad* components in the same workflow.
    - **bio.all.nc.model**: model trained on BioNLP-ST to identify uncertain events. Needed for *WEKA Classifier (uncertainty)* component. It is trained using a list of rules automatically extracted from BioNLP-ST using the lift formula to filter the meaningful rules (see Eq 3). The resulting uncertainty cue list, can be found in the *rule_resources* directory and should be used with both the *Rule Feature Generator Broad* and *Uncertainty Feature Generator Broad* components in the same workflow.
    - **combined.all.nc.model**: model trained on both GENIA-MK and BioNLP-ST to identify uncertain events. Needed for *WEKA Classifier (uncertainty)* component. It is trained using a list of rules automatically extracted from GENIA-MK and BioNLP-ST using the lift formula to filter the meaningful rules (see Eq 3). The resulting uncertainty cue list, can be found in the *rule_resources* directory and should be used with both the *Rule Feature Generator Broad* and *Uncertainty Feature Generator Broad* components in the same workflow.
8. **rule-cue_resources**:

- **cues.all.txt**: List of cues compiled based on GENIA-MK, BioNLP-ST and related literature, oriented to uncertainty in biomedical scientific publications.
- **rules.all.txt**: Rules extracted using the preselected biomedical list *cues.all.txt* using the *Uncertainty Rule Key Selection* component.
- **cues.ace.txt**: ACE-based (Thompson *et al.*, 2016) uncertainty cue-list.
- **rules.ace.txt**: Rules extracted using the preselected ACE-based list *cues.ace.txt* using the *Uncertainty Rule Key Selection Multiword* component.
- **rules.genia.nc.txt**: Rules extracted using *Uncertainty Rule Key Selection Broad (no cues)* component, by the GENIA-MK corpus.
- **rules.bio.nc.txt**: Rules extracted using *Uncertainty Rule Key Selection Broad (no cues)* component, by the BioNLP-ST corpus.
- **rules.combined.nc.txt**: Rules extracted using *Uncertainty Rule Key Selection Broad (no cues)* component, by the combination of GENIA-MK and BioNLP-ST corpora.

9. **DemoXMIout**: Empty directory to store XMI files written by the workflows

We advice the users to create their own directories and copy the files they need, especially if they intend to create/edit their own workflows on Argo or elsewhere. This way files will remain intact to be used by future potential users.

1.2 Description of end-to-end workflow for linking to pathway models

In this section, we describe the components that are necessary for the end-to-end workflow. Those components are used to annotate plain text sentences with events and uncertainty and then map them to model interactions and score the models. The workflow is available as a demo on Argo test server (full path: http://argo.nactem.ac.uk/test/?workflow=16863). In Figure 1 we present a conceptual schematic diagram of this workflow and its core processes. In the following sub-sections we elaborate on each conceptual block, discussing the components and parameters that we used for the implementation.

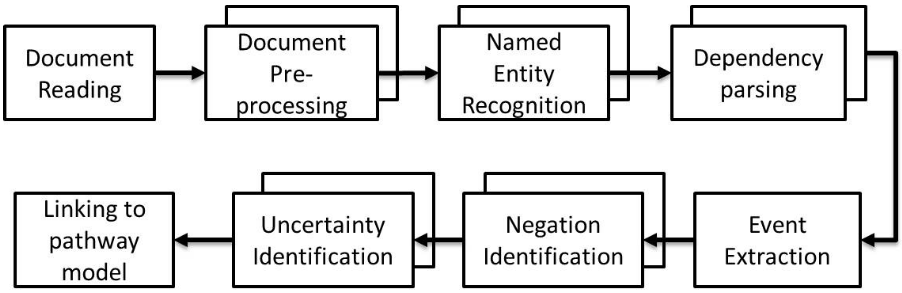

Figure 1: Conceptual schematic diagram of Argo workflow used for experiments. Tools that appear as multiple blocks, signify the use of multiple linked components in the actual workflow

1.2.1 Document Reading

The workflow begins with a document reader which loads a set of plain-text documents (usually from a remote server). In Argo there is a range of available document readers, that can parse different document formats, such as XML, XMI, TXT etc. For the presented workflow we used a TXT reader (*SFTP Document Reader* component), which reads one evidence passage per document, over an SFTP connection. The passages provided as evidence for the *B-cell Acute Lymphoblastic Leukemia Overview* pathway of Pathway Studio are already uploaded on the NaCTeM server under the name *leukemia_files*, as described in the beginning of the section. So are the sentences used for the Ras-Melanoma use-case, under the name *ras_melanoma_files*. Any other files, will have to be uploaded to a server and the server details will have to be set in the parameters.

The parameters to be set are the following:

- ServerURL: Server address
- RemoteDirectory: Path to the directory on the server
- Username: Username used to access the directory on the server

- Password: Password used to access the directory on the server
- Recurse: If set, implies recursive searching in the directory for files to read (not necessary for the default use-case)

1.2.2 Document Pre-processing

After reading plain documents, a set of pre-processing steps are necessary in order to prepare the text for core NLP tasks such as named entity recognition and event extraction. The *Regex Annotator* separates text in paragraphs identifying *end-of-line* characters. Following paragraph segmentation, the text is segmented into sentences. For all our experiments, we used the LingPipe Sentence Splitter (Alias-i, 2008). The sentences are then decomposed into chunks and tokens. *OSCAR 4 Tokeniser* is used for tokenising (Jessop *et al.*, 2011) while the GENIA Tagger (Tsuruoka *et al.*, 2005) is used for chunking as well as lemmatisation and part-of-speech (POS) tagging. The lemmata and POS tags produced are used in the named entity recognition task as features.

1.2.3 Named Entity Recognition

The next step is Named Entity Recognition (NER). As this step is crucial for efficient event extraction and the subsequent uncertainty annotation, it is imperative to identify different named entities (proteins, drugs, cells) that participate in events with high accuracy. Thus a series of components are used in order to efficiently extract different NE types, as each component specialises in certain entity type(s). An *Annotation Remover* component is used in certain cases after NER components to filter annotations for particular entity types that are redundant or not needed in this application. *Annotation Remover* components are also used to remove features that were generated for particular components and need not be used by the components that follow. Essentially the Annotation Remover components are used in any case when a specific type of CAS annotation needs to be removed. The type(s) of annotation to be removed can be chosen in the parameters.

The *Genia Tagger* component used for chunking also extracts the *Cell Line* entities. It produces some protein entity annotations as well, that are removed by the *Annotation Remover* component that follows. Subsequently, *Protein* entities are extracted using a NERsuite based model trained for protein identification (*NERsuite Dictionary Feature Generator* and *NERsuite Tagger* components). The *Annotation Remover* that follows serves to remove the generated features. Next, the *NERsuite Custom Tagger* uses a NERsuite model that uses dictionary features and is trained on BioCreative II GM Track training corpus[2] in order to identify *Gene* and *Gene_product* entities. The two *Overlapping Annotation Removers* merge the overlapping *Gene* and *Protein* annotations to maintain only the *Protein* ones, using exact and relaxed string matching respectively. The overlapping annotations of Genes, Proteins and Protein Family annotations are further disambiguated and resolved by the *Gene/Protein Family Disambiguator* component. The filtered annotations are then used as input for the *Similar Text Span Annotator* components.

Having finished with protein and gene related annotations, the *Chemical Entity Recogniser*, *Type Mapper*[3] and *Similar Text Span Annotator* components that follow are used to generate *Chemical* and *Drug* entity annotations.

The *Concept Normaliser* components provide grounding for the identified *Protein* and *Chemical* entities to the UniProt[4] and ChEBI[5] database indexing respectively. Two *NERsuite Custom Tagger* components follow with models trained on the BioNLP 2013 Pathway Curation corpus Nédellec *et al.* (2013). The former identifies protein complexes, while the latter identifies subcellular locations.

The components that follow perform mainly filtering and disambiguation tasks that resolve overlaps and annotation conflicts, or correct annotation spans. More specifically, the *Function Word Annotator* components extend already identified annotation spans for different entity types to include the term "inhibitor". A series of *Overlapping Annotation Remover* components resolve overlaps for different entity pairs (Chemical - Gene, Cell-line - Gene, Cell-line - Chemical, GeneOrProtein - Protein Family).

1.2.4 Dependency Parsing

Subsequently syntactic dependencies are extracted using the Genia Dependency Parser [6] as well as the Enju dependency parser (Matsuzaki and Tsujii, 2008) components. Both components generate annotations for dependencies that are used by the *EventMine* component that follows. Moreover, the dependencies generated by Enju are also used by the negation and uncertainty identification components. Enju also produces Part-of-Speech

[2]http://www.biocreative.org

[3] *Type Mapper* components are used throughout the workflow to map annotations among different TypeSets used by different components

[4]http://www.uniprot.org/

[5]https://www.ebi.ac.uk/chebi/

[6]http://people.ict.usc.edu/ sagae/parser/gdep/

(POS) and lemmatising annotations, that are used as features in the subsequent components (*EventMine* and *Feature Generator* components).

1.2.5 Event Extraction

For the event extraction that follows we use EventMine trained on multiple corpora using the wide coverage approach described by Miwa *et al.* (2013). The corresponding argo component used for this purpose is called *EventMine for BioNLP Shared Tasks.* For the wide coverage approach to be used, the *multi-event* value has to be chosen in the *model* parameter. If the events are to be mapped to model interactions they can be further optimised at the last step of the workflow, towards matching the interactions of the pathway.

1.2.6 Negation Identification

The negation identification components are implemented on the basis of a negation identification module inspired by a machine learning method described by Nawaz *et al.* (2013), complemented with a rule based component. The machine learning part is realised with *Event Feature Generation* and *Negation Feature Generation* in terms of the feature generation components. Then *WEKA Classifier* can be used with the negation model provided in resources. For the correct configuration of the *WEKA Classifier*, the *Target Feature* option has to be set to *attributeValue.* The rule-based component is instantiated with the *Negation Rule Checker.*

1.2.7 Uncertainty Identification

For the uncertainty identification task, the workflow presented uses a random forest classifier implemented using the WEKA API (Hall *et al.*, 2009). In the demonstration workflow the incorporated components use the preselected uncertainty cue list that was compiled based on GENIA-MK and BioNLP-ST annotations as well as related work on uncertainty for scientific literature. Also, a simple Rule Checker component is added in the workflow (*Uncertainty Rule Checker*). Alternative options would be to swap the components and use the ACE-based list of cues, or the automatically generated list of cues. Different feature extraction components need to be used in each case for the related model to run properly. In all cases the final model is a Random Forest classifier that needs to be used in the *ModelFile* parameter in the *WEKA classifier* component. The other parameter to set in the *WEKA classifier* is the *TargetFeature* that needs to be set to *attributeValue.* We describe below the necessary components depending on the kind of cue approach in order to allow the users to appropriately edit the workflow. Note that the order of the feature extraction components will not affect the output of the classifier.

1. For the use of the rules generated with the use of the preselected uncertainty cue list the necessary components are:
   - The *Event Feature Generator* component necessary for event-based features. This component is not affected by the cue selection and does not need any parameters to be set.
   - The *Rule Feature Generator* component necessary for the rule-based features. The rules extracted using the preselected uncertainty cue list need to be set in the *RuleFile* parameter (see Section 1.1).
   - The *Uncertainty Feature Generator* component is necessary for the rest of the features that are related to uncertainty cues. This component takes as input a list of cues (i.e. preselected uncertainty cue list) as the *cueList* parameter (see Section 1.1).
2. For the use of the rules generated with the use of the ACE uncertainty cue list the necessary components are:
   - The *Event Feature Generator* component necessary for event-based features. This component is not affected by the cue selection and does not need any parameters to be set.
   - The *Rule Feature Generator ACE (multiword)* component is necessary for the rule-based features as it can account for multi word cues in the ACE-based cue list. The rules extracted using the ACE-based list need to be set in the *RuleFile* parameter (see Section 1.1).
   - The *Uncertainty Feature Generator ACE (multiword)* component is necessary for the rest of the features that are related to uncertainty cues. Similarly to the rule feature generator coponent described above, it can account for multi-word cues. This component takes as input a list of (potentially multiword) cues (i.e. ACE based list) as the *cueList* parameter (see Section 1.1).
3. For the use of the automatically generated rule list the necessary components are:
   - The *Event Feature Generator* component necessary for event-based features. This component is not affected by the cue selection and does not need any parameters to be set.

- The *Rule Feature Generator* component necessary for the rule-based features. The file containing the list of rules need to be set in the *RuleFile* parameter (see Section 1.1).
- The *Uncertainty Feature Generator Broad* component is necessary for the rest of the features that are related to uncertainty cues. This component takes as input a rule list and automatically generates the cues from it. In order to avoid noisy cue generation it requires a list of stop -words to be provided in the *stopWord* parameter, and the set of rules to be provided in the *ruleList* parameter (see Section 1.1).

It should be noted that the order of the feature extraction components will not affect the output of the classifier.

1.2.8 Linking to the model

The component to be used in order to link evidence passages and associated evidence to a pathway or interaction network, depends on the encoding/format of the network. Currently there are two components available that support two different formats, namely Pathway Studio data in TSV and BioPAX data in JSON format.

- *Uncertainty On Pathway Models (PS version)*: This model supports Pathway Studio data (Nikitin *et al.*, 2003) extracted in TSV format and was used for the Leukemia model use-case presented in the main manuscript, Section 4.2.1. This component:

  1. Checks each interaction of the pathway against the extracted events from the literature (also optimising the extracted events to match the pathway interactions using an approach adapted from Zerva and Ananiadou (2015).
  2. Calculates the score of the interaction based on the uncertainty of the related events as indicated in the formula of Equation 6 in Section 3.3 of the main manuscript.

  It requires setting the following parameters:

  – *InputFile:* Path to the uploaded Pathway Studio .csv file. Default file: "leukemia.csv". Pathway files can be downloaded from Pathway Studio using the export option.

  – *OutputFile:* Path to the output Pathway Studio .csv file, that will include the uncertainty values and scores. It will append two columns at the end of the initial csv file: "Uncertainty Score" and "Uncertain Event Percentage". The former represents the score as described in the main paper, and the latter the percentage of identified uncertain events over the total of events mapped to the interaction. Default value: leukemia_uncertain.csv

  – *DepDepth:* The threshold value for the dependency depth in order to link missed arguments. Default value: 3

  – *RelationMatch:* Set to true for a strict matching of relation types. Default value: false

  – *alpha:* Allows setting the alpha value (see Equation 4 and 5 in the main manuscript). The default is 0.5

- *Evidence Linking to Pathways (BioPAX)*: This model supports BioPAX models in JSON format and was used for the Ras-Melanoma use-case described in Section 4.2.2 of the main manuscript. This component:

  1. Checks each node (interaction and entities) of the pathway against the extracted events from the literature.
  2. Calculates the score of the interaction based on the uncertainty of the related events as indicated in the formula of Equation 6 in Section 3.3 of the main document.

  It requires setting the following parameters:

  – *InputFile:* Path to the initial BioPAX model encoded in JSON format. Ras.json in resources/ras_models fits this description.

  – *OutputFile:* Path to the output JSON file, that will include the linked sentences, uncertainty values and scores in the JSON format. Sample output: Ras-melanoma.json.

  – *alpha:* Allows setting the alpha value (see Equations 4 and 5 in the main manuscript). The default is 0.5

  – *strict_match:* Set to true for a strict matching of relation types. Default value: false

1.2.9 Other outputs

Instead of linking to the model, the annotated sentences, can be visualised using the *Manual Annotation Editor* component or written to files and stored on a server using an *SFTP XMI Writer* component. In the case of the *Manual Annotation Editor*, the annotation types to be visualised have to be set in the parameter settings of the component (tick the corresponding boxes on the *Parameters* tab). By default we have enabled: *CancerMechanisms, BioNLP-ST-Typesystem.*

Alternatively the processed files can also be written on a server in XMI format, using the *SFTP XMI Writer*. In this case the following parameters have to be set:

- Server: Server address
- RemoteDirectory: Full path to the directory on the server used to output files
- Username: Username used to access the directory on the server
- Password: Password used to access the directory on the server
- Port: Set to *22* by default
- RecorderEnabled: Set to *False* by default

Finally, the output files can also be written in BioNLP standoff format (see Section 1.3.1 and Figure 4 for details on the format) and visualised on Brat (Stenetorp *et al.*, 2012). The *BioNLP ST Data Writer* can be used for this reason, but the files will have to be stored on the Argo server. The user will have to create a folder in the *Documents* tab and then select the name of the folder in the settings (*OutputFolder*). The *BioNLP ST Data Writer* component is not added in the public workflow, but it is available as a Consumer-type component on the Editing panel, and can be added to the workflow by the user (see Section 1.2.10 that follows for instructions on how to edit a workflow).

1.2.10 Full workflow and access instructions

The full workflow as viewed when editing the *"DEMO of end-to-end linking textual uncertainty to pathway interactions"* public workflow is presented in Figure 2.

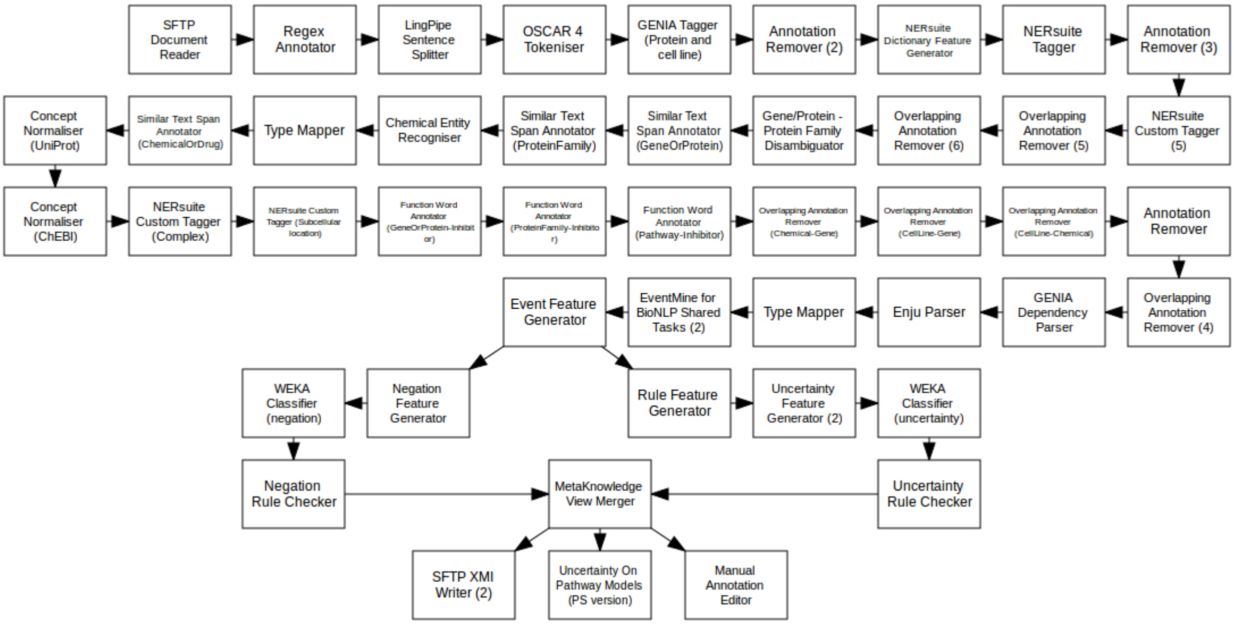

Figure 2: End-to-end Argo workflow used for experiments on linking evidence to a pathway model using uncertainty as a scoring criterion.

To access the workflow [7] visit http://argo.nactem.ac.uk/test/, select this workflow and select *Edit* or *Run* among the options that appear above the workflow list. In order to make changes to any of the parameters and components, it is necessary to make a new copy of the workflow. This can be achieved by selecting *Edit* and subsequently selecting *More > Save as Copy.*

[7] Full path to the exact workflow : http://argo.nactem.ac.uk/test/?workflow=16863

1.3 Description of workflow processing large corpora with binary uncertainty

In this section we provide the description of a workflow that can process corpora annotated with stand-off annotations for events and named-entities, in order to identify event uncertainty, as described in the main manuscript.

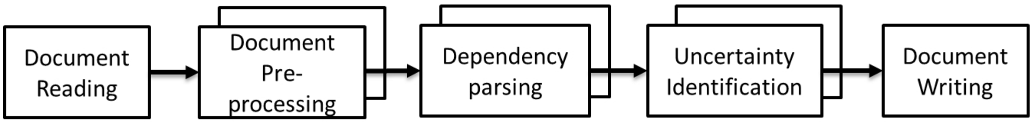

Figure 3: Conceptual schematic diagram of Argo workflow used for experiments. Tools that appear as multiple blocks, signify the use of multiple linked components in the actual workflow

1.3.1 Document Reading

For the experiments with pre-annotated corpora with events, it is necessary to use one of the BioNLP document readers. We have used the *SFTP BioNLP Shared Task Data Provider* that allows users to read files over an SFTP connection. Files have to follow specific standoff format; annotations are stored in two files file (.a1, .a2) separately from the annotated document text that is stored in one .txt file. All three files must be identically named. The annotation files have to follow the structure depicted in Figure 4. Annotations referring to the entities are stored in .a1 files while annotations referring to the events, stored in the .a2 files[8]. The Genia-MK and BioNLP-ST annotations are already uploaded on the NaCTeM server as explained in the beginning of Section 1. Any other files, will have to be uploaded to the same server or some other and the server details will have to be set in the parameters. Alternatively, the *SFTP BioNLP Shared Task Data Provider* can be swapped for *SFTP BioNLP ST Data Reader* and the files can be uploaded on the Argo server.

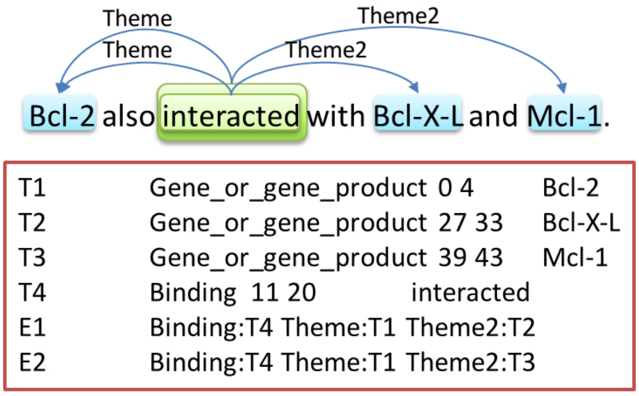

Figure 4: Standoff annotation example: T# denotes entities, and E# events. The type of the entity follows in the same line separated with a tab, and followed by the offsets of the corresponding terms in the sentence. Then the exact text extract follows also separated by a tab. Note that event triggers are also annotated as entities. For events, there is a tab separated entry indicating Event trigger and arguments with their roles. All triggers and arguments must be annotated in the sentence.

1.3.2 Document pre-processing and Dependency parsing

The pre-processing part starts with text being segmented into sentences. For all our experiments, we used LingPipe Sentence Splitter (Alias-i, 2008). The sentences are then decomposed into chunks and then tokens. For this purpose, we used GENIA Tagger (Tsuruoka *et al.*, 2005). Then syntactic dependencies are extracted using Enju dependency parser (Matsuzaki and Tsujii, 2008), which also performs lemmatization and part-of-speech tagging. The lemmas and POS tags are used subsequently by the feature extracting components for uncertainty identification.

[8]For further details on the annotation structure see: http://2011.bionlp-st.org/home/file-formats

1.3.3 Uncertainty Identification

For the uncertainty identification task, we used the random forest classifier implemented using the WEKA API as we did for the workflow described in Section 1.2. The provided model has been trained using rule, event and cue based features described in the main document. Event based features are extracted using the "*Event Feature Generator*" component, the rule based features are extracted using the "*Rule Feature Generator*" one, and the rest of the features that are related to uncertainty cues, are extracted by the use of "*Uncertainty Feature Generator*". The order of the feature extraction components will not affect the output of the classifier. The "*WEKA classifier*" component (Hall *et al.*, 2009) will then use the extracted features and the trained model (assigned in the *ModelFile* parameter). It should be noted that different combinations of the feature generator components are available based on the descriptions in Section 1.2.7.

1.3.4 Saving/displaying results

The produced uncertainty annotations, can either be viewed using the *Manual Anntoation Editor* component or written to a .tsv file using the *Event Listing* component. The .tsv file has the following fields: *Event ID, Event type, Covered text (by trigger), Paper ID, sentence (full sentence text), Certainty level (for GENIA), KT (for GENIA), Speculation, Uncertainty (value attributed by our tool).* For this component the following parameters have to be set:

- OutputFile: The name of the file to list the events and uncertainty information in the output
- KT : If using on Genia-MK and not on BioNLP-ST data, setting KT to true, will also output the original, knowledge type (KT) annotations for comparison reasons.

Alternatively annotated files can also be written on a server in XMI format, using the *SFTP XMI Writer*. In this case the following parameters have to be set:

- Server: Server address
- RemoteDirectory: Full path to the directory on the server used to output files
- Username: Username used to access the directory on the server
- Password: Password used to access the directory on the server
- Port: Set to *22* by default
- Recorder: Set to *False* by default recorderEnabled

Finally, as in the workflow presented in Section 1.2 results can be written in BioNLP standoff format and visualised on Brat (Stenetorp *et al.*, 2012). The *BioNLP ST Data Writer* can be used for this reason, but the files will have to be stored on the Argo server. The user will have to create a folder and then select the name of the folder in the settings (*OutputFolder*). The *BioNLP ST Data Writer* component is not added in the public workflow, but it is available as a Consumer-type component on the Editing panel, and can be added to the workflow by the user (see Section 1.3.5 that follows for instructions on how to edit a workflow).

1.3.5 Full workflow and access instructions

The full workflow as viewed when editing the "DEMO Uncertainty tagging on event annotated corpora" public workflow is presented in Figure 5.

The process of accessing the workflow is similar to the one described in Section 1.2.10. To access the workflow visit http://argo.nactem.ac.uk/test/ (Full path: http://argo.nactem.ac.uk/test/?workflow=16878), select this workflow and select *Edit* or *Run* among the options that appear above the workflow list. In order to make changes to any of the parameters and components, it is necessary to make a new copy of the workflow. This can be achieved by selecting *Edit* and subsequently selecting *More* and then *Save as Copy.*

1.4 Description of additional related components

In this section we describe components available on Argo platform that do not participate in the previously described workflows, but are crucial for training models and extracting rules.

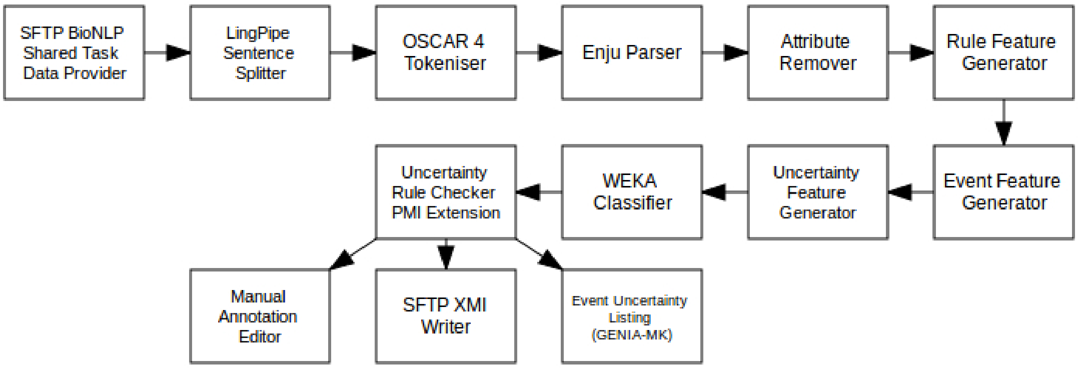

Figure 5: End-to-end Argo workflow used for experiments on annotating large corpora with binary uncertainty values.

WEKA Trainer component

All models described in Section 1.1 have been trained using *WEKA Trainer* component. For a model to be trained, a set of *Feature Extractor* components have to precede it in the workflow. The same feature extractor components need to be used when using the model with the *WEKA Classifier* component in order to properly classify the instances that need to be annotated. The order of the *Feature Extractor* components will not influence the results. We describe below the parameter configurations for this component:

- **Algorithm:** The classifier algorithm to be used. Choose RandomForest.
- **ModelFile:** The full path to ne model to be created. For example : "My Documents/models/modelName.mdl". Click on *Select* in order to choose the appropriate path, and enter the desired model name in the white dialog box.
- **ParameterString:** Here the training parameters for the WEKA trainer can be set. Allows to set parameters like number of iterations, depth of tree, minimum variance for split etc. The full list of optinos can be accessed here: `http://weka.sourceforge.net/doc.dev/weka/classifiers/trees/RandomForest.html`. For the experiments described in the main manuscript we use: "-I 100 -K 0 -S 1 -num-slots 1".

In order to use the *WEKA Trainer* component to train model for uncertainty identification on events, the presence of a corpus with uncertainty annotated events is necessary. Also, pre-processing the corpus with NLP procedures such as tokenisation and dependency parsing is necessary for most of the *Feature Generator* components. Assuming existence of an event and uncertainty annotated corpus like the GENIA-MK or the BioNLP-ST, a sample component sequence for a model training workflow would be: *SFTP BioNLP Shared Task Data Provider > LingPipe Sentence Splitter > OSCAR 4 Tokeniser > Enju Parser > Event Feature Generator > Uncertainty Feature Generator > WEKA Trainer.*

Uncertainty Rule Key Selection components

Uncertainty Rule Key Selection components are used to extract rule patterns used by the *Rule Checker* and the *Rule Feature Generator* components. Below we describe the different types of *Rule Key Extraction* components that are available on Argo test server.

- **Uncertainty Rule Key Selection:** It is the simplest among the rule-pattern extraction components presented in this document. It takes a list of (ideally single word) cues as input for the *CueFile* parameter. The list has to be in TXT format, with one cue per line.
- **Uncertainty Rule Key Selection Multiword:** This component has the same functionalities as the simple *Uncertainty Rule Key Selection*, but can better accommodate multiword cues, when searching for dependency paths. This component was used for the experiments using cues from the ACE corpus (Thompson *et al.*, 2016), since the list of cues annotated for this corpus contained multiword expressions of uncertainty.

- **Uncertainty Rule Key Selection Broad (no cues):** This component allows for rule pattern extraction without the need of a cue list to guide the pattern generation. It will generate all potential 1-hop and 2-hop dependency patterns around uncertain events and then filter them according to the thresholds given for one or more of the following parameters: *LiftFilter, LeverageFilter, JaccardFilter, JmeasureFilter*. Each parameter corresponds to the Equations 3 – 6 described in Section 3.2.2.

The generated rule patterns will be of the following format:

- 1-hop dependencies: <word>_D0_<dependency type>
- 2-hop dependencies: <word 1>_<word 2>_D1_<dependency type 1>_<dependency type 2>

where the <word> part corresponds to the words being part of the pattern and the <dependency type > part can belong to one of the four core dependency types of *Enju Parser*, namely $[ARG1, ARG2, ARG3, ARG4, MOD]$. ARG1 denotes a subject of a verb, a target of modification by modifiers, etc. ARG2 corresponds to object of verbs, prepositions, etc. ARG3 and ARG4 are objects and complements of verbs, etc. Finally, MOD stands for participial constructions and denotes a clause modified by another clause, if the subordinate clause has an ARG1.

All Rule Key Selection components assume presence of uncertainty annotated events, in order for the rule extraction procedure to function properly. Also, since it rule extraction is based on dependency patterns, *Enju Parser* should also be used. Assuming an event and uncertainty annotated corpus like the GENIA-MK or the BioNLP-ST, a typical/minimal component sequence for a rule extraction workflow would be: *SFTP BioNLP Shared Task Data Provider > LingPipe Sentence Splitter > OSCAR 4 Tokeniser > Enju Parser > Uncertainty Rule Key Selection* .

2 Classification of uncertainty and coverage in various corpora

As explained in the main document, we aimed for a broad coverage of diverse uncertainty expressions, mentioned in previous work, that we categorised, based on they way they are expressed as shown in Figure 3 of the main document. Below we provide a description of each category.

1. Speculation: Claims where the author expresses speculation, hedging or hesitation. It can be further analysed into:
   (a) Strong Speculation: Words such as may, might, perhaps.
   (b) Weak Speculation: words such as suggest, indicate, etc., that, while they are not as strong as in (a), they are still distinct in terms of the conveyed certainty when compared to words such as prove, show, etc.
2. Investigation: Events or claims that are mentioned in the context of an investigation (experiment, test, analysis).
3. Admission of lack of knowledge (AOLK): Cases where the author acknowledges an as yet unknown or partially known event.
4. Relays of external hypotheses (weaseling): Cases where the author mentions something as a fact without clearly indicating the source (using expressions such as it is believed to be, it is known, etc.).
5. Time or frequency concerns: Cases where the event seems to occur with limited frequency, as indicated by expressions such as usually, sometimes, etc.

In Table 1, we summarise the annotation schema and coverage of the above aspects among available, annotated corpora.

Table 1: Coverage of different uncertainty types by available biomedical corpora

	Corpus	BioScope	BioNLP-ST	CoNLL Bio-task	Genia-MK
	Annotation level	sentence	event	sentence	event
	Classification	binary	binary	binary	3-class
	Passage type	abstracts + full papers	abstracts + full papers	abstracts + full papers	abstracts
Uncertainty	Speculation (Strong)	**YES**	**YES**	**YES**	**YES**
	Speculation (Weak)	**YES**	**NO**	**YES**	**YES**
	Investigation	**NO**	**NO**	**NO**	**YES**
	AOLK	**YES**	**YES**	**YES**	**NO**
	Weaseling	**NO**	**NO**	**NO**	**YES**
	Limited Frequency	**NO**	**NO**	**NO**	**NO**

3 Details on implementation of the hybrid model

3.1 Machine Learning

3.1.1 Additional features in the machine learning component

In this section we present a comprehensive table of additional features that were implemented and used in the Random Forest classifier models, along with the rule-based features described in the main manuscript, Section 3.2. The features presented in Table 2 are used in the models that were generated for all the experiments presented in Section 4 of the main manuscript.

Table 2: Features used for uncertainty detection

Feature Category	Corresponding Argo Component	Sub-category	Feature	Output
Event	Event Feature Generator	Lexical	Event-trigger surface form	Nominal
			POS tags	Nominal
		Semantic	Event type	Nominal
			Argument type	Nominal
			Argument role	Nominal
		Complexity	Complex/simple	Binary
Cue	Uncertainty Feature Generator	Lexical	Existence of cue	Binary
			Cue surface form	Nominal
			POS tags	Nominal
Event & Cue	Uncertainty Feature Generator	Relative position	#words between cue and trigger	Numeric
			Position of cue before/after trigger	Binary
		Dependency	Direct dependency between cue and trigger	Binary
			Shortest dependency path length	Numeric
		Constituency (syntactic)	Command[9] of cue over trigger	Binary
			Command of cue over arguments	Binary

3.2 Rule induction

In this section we present further details related to the rule induction procedure. In Section 3.2.1 we present an example of rule extraction and application. In Section 3.2.2 we present more details and evidence for our choices in terms of constraining and filtering the extracted rule-sets.

[9]Command of a word a over word b, signifies that in the syntactic tree a is the head of a branch that contains b

3.2.1 Example of rule extraction and application

In the main manuscript we describe the formulation of dependency rules as chains of words and dependencies (see Section 3.2, Equations 1-3 in the main manuscript). In Figure 6 we present a step-by-step example of the rule extraction procedure as well as the procedure of mapping the same rule to a new "unseen" sentence.

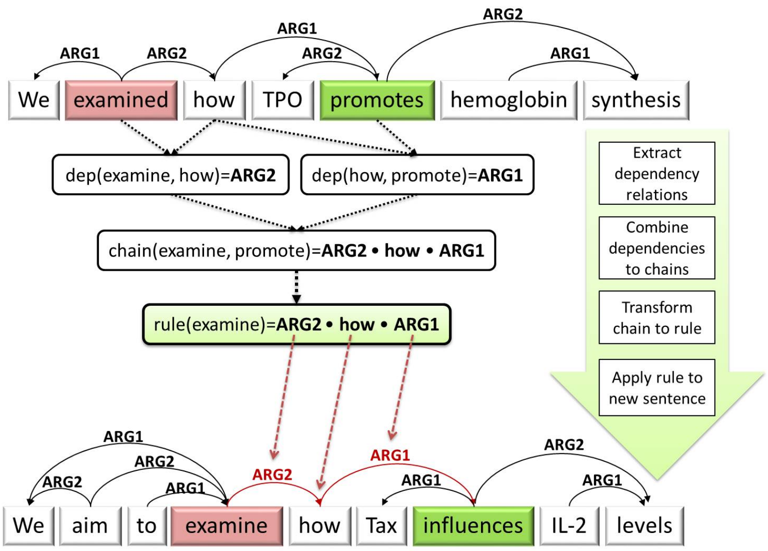

Figure 6: Illustration of rule induction steps from a sentence (top) and application (bottom) to a new sentence to be annotated.

In the top sentence, we observe the dependency relations that are extracted as a first step (using Enju (Matsuzaki and Tsujii, 2008) dependency parser). Subsequently, the ARG2 dependency between *examine* and *how* and the ARG1 dependency between *how* and *promotes* are combined into a $chain(T_S, T_T)$ relation, where T_S is the cue *examine* and T_T the event trigger *promote*. Since *promote* is an event trigger, we get a new rule pattern for the cue *examine* ($ARG2 \cdot how \cdot ARG1$). The pattern can then be applied to any sentence where the same dependency path exists between the cue *examine* and any event trigger.

3.2.2 Constraining and filtering rule patterns

As stated in the main manuscript, it is important to limit the initially extracted set of rule patterns. This will help decrease the search space when it comes to rules applied on their own, or the feature space when it comes to rule patterns being used as features for our machine learning component.

Moreover, it is important to limit the pattern set while maintaining the more meaningful and relevant rules in order to boost recall without compromising precision. The latter is particularly important in those cases that do not use a list with uncertainty cues to limit the rule pattern generation. In those cases, any dependency chains that have one end on an uncertain event trigger would be extracted as potential rules. As such, filtering them in terms of informativeness is necessary to avoid a noisy rule-set.

3.2.2.1 Filtering pattern-set with measures of informativeness

One or more measures of informativeness can be used to filter the potential rule patterns and retain only the most meaningful ones. We have experimented with the measures commonly used for mining association rules, as we can regard our problem as an association rule problem of the form $X \Rightarrow Y$, where X is the rule pattern and Y is uncertainty. Most association rule measures are based on the support-confidence framework presented in Equations 1 and 2.

$$supp(X \Rightarrow Y) = supp(X \cup Y) = P(X \wedge Y) \tag{1}$$

$$conf(X \Rightarrow Y) = \frac{supp(X \Rightarrow Y)}{supp(X)} = \frac{supp(X \cup Y)}{supp(X)} = \frac{P(X \wedge Y)}{P(X)} = P(Y|X) \tag{2}$$

We experimented with Interest (also called Lift) (Brin *et al.*, 1997), Leverage (Piatetsky-Shapiro, 1991), Jaccard similarity (Tan *et al.*, 2004) and J-measure (Smyth, 1991), as presented in Equations 3 - 6.

$$interest(X \Rightarrow Y) = \frac{conf(X \Rightarrow Y)}{supp(Y)} = \frac{P(X \wedge Y)}{P(X) \cdot P(Y)} \tag{3}$$

$$leverage(X \Rightarrow Y) = supp(X \Rightarrow Y) - supp(X) \cdot supp(Y) = P(X \wedge Y) - P(X) \cdot P(Y) \quad (4)$$

$$jaccard(X \Rightarrow Y) = \frac{supp(X \cup Y)}{supp(X) + supp(Y) - supp(X \cup Y)} = \frac{P(X \wedge Y)}{P(X) + P(Y) - P(X \wedge Y)} \quad (5)$$

$$jmeasure(X \Rightarrow Y) = P(X \wedge Y) \cdot log(\frac{P(Y|X)}{P(Y)} + P(X \wedge \bar{Y}) \cdot log(\frac{P(\bar{Y}|X)}{P(\bar{Y})} \quad (6)$$

We then applied them on a random subset of BioNLP-ST and GENIA-MK, without limiting the rule extraction with any lists of cues. We obtained a set of 500 rule patterns, and manually classified them in five categories: *Certain*, *Dubious*, *Irrelevant*, *Stopwords*, *Uncertain*. We defined each category as:

- Certain: Rule that contains at least one term that is an indicator of certainty (eg. cues such as "prove", "confirm" etc.)
- Dubious: Rule that has a cue that could be related to uncertainty under conditions, such as "particularly", "how" etc.
- Stopwords: Rule that contains solely stopwords (The stopword list was compiled based on `http://www.ranks.nl/stopwords`)
- Uncertain: Rule that contains at least one term that is an indicator of uncertainty (eg. cues such as "may", "likely" etc.)
- Irrelevant: Rule that does not fall under any of the above categories. Usually they contain biomedical terms, found in the vicinity of event triggers, but not part of them, such as "synergistic", "angiogenic" etc.

We then applied the measures described above using the uncertainty annotations by the BioNLP-ST and GENIA-MK corpora and we plotted the results for each measure as shown in Figures 7-10.

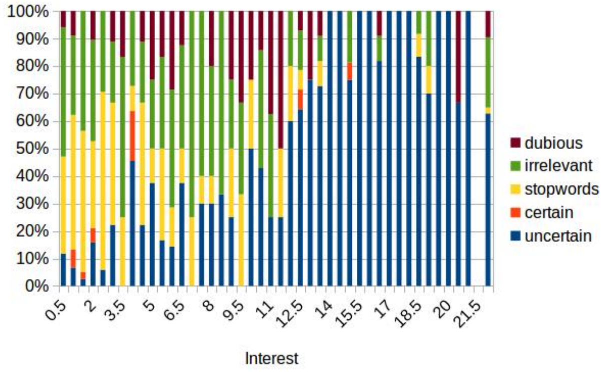

Figure 7: Coverage of different pattern categories based on Interest (Lift) value

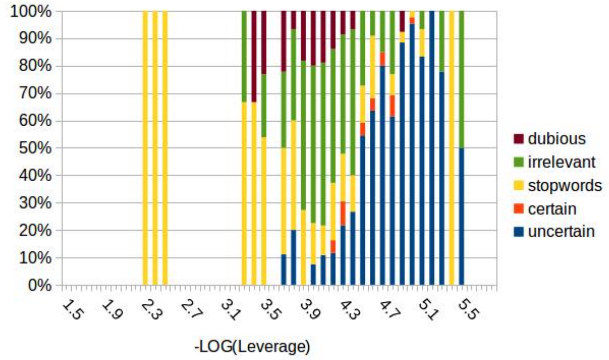

Figure 8: Coverage of different pattern categories based on Leverage value

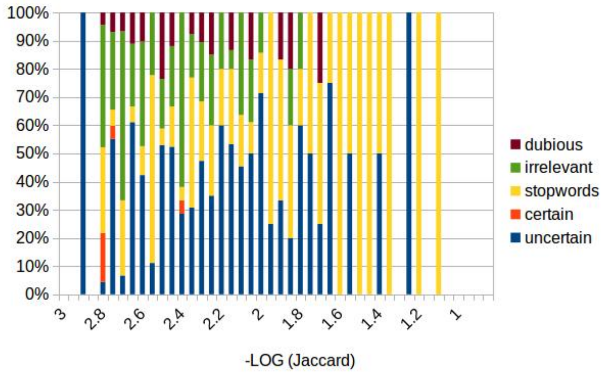

Figure 9: Coverage of different pattern categories based on Jaccard value

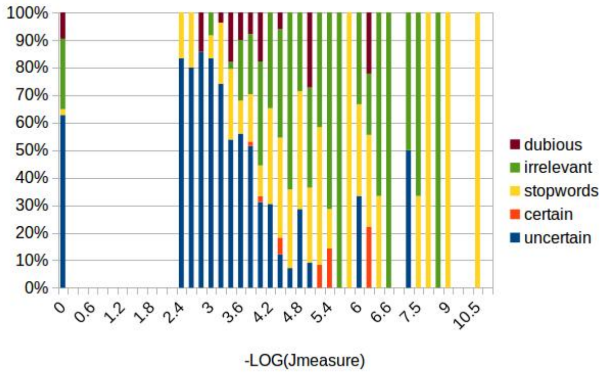

Figure 10: Coverage of different pattern categories based on J-measure value

We also considered combinations of measures, but no combination of thresholds seemed to better discriminate uncertain rules from the rest in a statistically significant way. Based on Figures 7–10 we decided that the use of *Interest* was sufficiently discriminative as a filter for our experiments. Indeed as we can see in Figure 7, by choosing a threshold between 11 and 12 we can get the majority of uncertainty related cues, avoiding most of the "stopwords" and "irrelevant" ones.

3.2.2.2 Limiting pattern-set by constraining length of dependency chains

As stated in the main document, Section 3.2.1, rule-patterns used in order to identify uncertain events, are essentially dependency chains ($chain()$ function as presented in Equation 2), that capture existing dependency paths between uncertainty cues and event trigger words. These patterns are extracted by corpora containing bio-events annotated with uncertainty. During the rule-pattern extraction process, the search space and computational complexity constitute an important concern pertaining to the robustness of the system. The chain length can significantly impact the search space. Assuming the maximum allowed chain length (# of tokens w) is n, all w come from a dictionary size $|D|$ and the size of the dependency output set is S, the upper bound on the generated number of patterns can be calculated using multiset coefficients as in Equation 7.

$$|PatternSet| = \sum_{k=0}^{n} \frac{(D+k+1)! \cdot (S+k+1)!}{(-k-1)! \cdot k!} \quad (7)$$

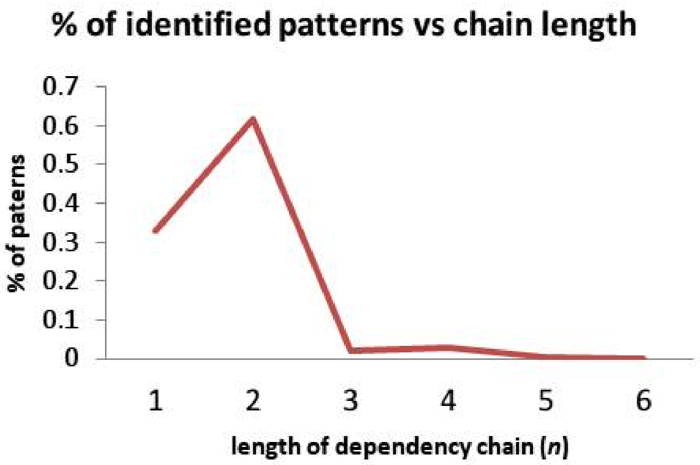

Figure 11: Percentage of identified patterns for increasing n (chain length) on Genia-MK. We can observe that the percentage converges to 0 for $n > 2$.

While the size of the dictionary $|D|$ could not be constrained, we estimated that we can retrieve more than 95% of the potential uncertainty patterns over a corpus while constraining chain length to be $n < 3$, as indicated in Figure 11.

4 Use-cases

4.1 Leukemia use-case: Details and expansion

4.1.1 Inter-Annotator agreement

In this section we present the inter-annotator agreement for the annotations of the Leukemia model use-case as presented in Section 4.2.1. The agreements calculated using the Cohen's kappa value Cohen and Jacob (1968) for each pair of annotators.

4.1.2 Second annotation attempt with quantitative scoring

Following up on the results presented for the Ras-melanoma model in the main manuscript (see Section 4.2.2) we have repeated the annotation process for the full set of sentences and interactions of the Leukemia model.

In a similar fashion to the Ras-melanoma annotation procedure, annotators were presented with 72 interactions and the passages that were linked to them. The events identified by EventMine (Miwa *et al.*, 2012),

Table 3: Inter-annotator agreement for each annotator pair (Kappa value)

	A1	A2	A3	A4	A5	A6	A7
A1	1	0.52	0.69	0.82	0.77	0.59	0.61
A2	0.52	1	0.53	0.66	0.53	0.59	0.60
A3	0.69	0.53	1	0.66	0.65	0.62	0.59
A4	0.82	0.66	0.66	1	0.83	0.67	0.62
A5	0.77	0.53	0.65	0.83	1	0.73	0.64
A6	0.59	0.59	0.62	0.67	0.73	1	0.68
A7	0.61	0.60	0.59	0.62	0.64	0.68	1

were annotated for each passage, but the decision related to the (un)certainty of each event was not revealed. Similarly, there was no indication of the score attributed by our system for the interaction in question. Five users[10] were asked to study each passage and score the annotated event based on uncertainty, on a 1-5 scale. Upon scoring all passages accompanying an interaction, they were asked to score the interaction, also on a 1-5 scale, based on the passages they just annotated.

The distribution of scores for the passage annotations are shown in figure 12, and we can observe, that while generally most of the events are allocated high certainty scores by all users, there seem to be different scoring patterns for some users. For example annotator #3 seems to attribute most of the events with a score of 3-4 as opposed to most of the annotators that tend to attribute the greatest percentage of events with 4-5 scores. On the contrary, annotator #4 identifies considerably more events as fully uncertain (score of 1). Compared to the Ras-melanoma experiment described in the main manuscript, Section 4.2 where we had only 2 annotators, we can see that in this case, there is increased diversity in the perception of textual uncertainty. This observation further encourages future work on user-adaptive identification of uncertainty.

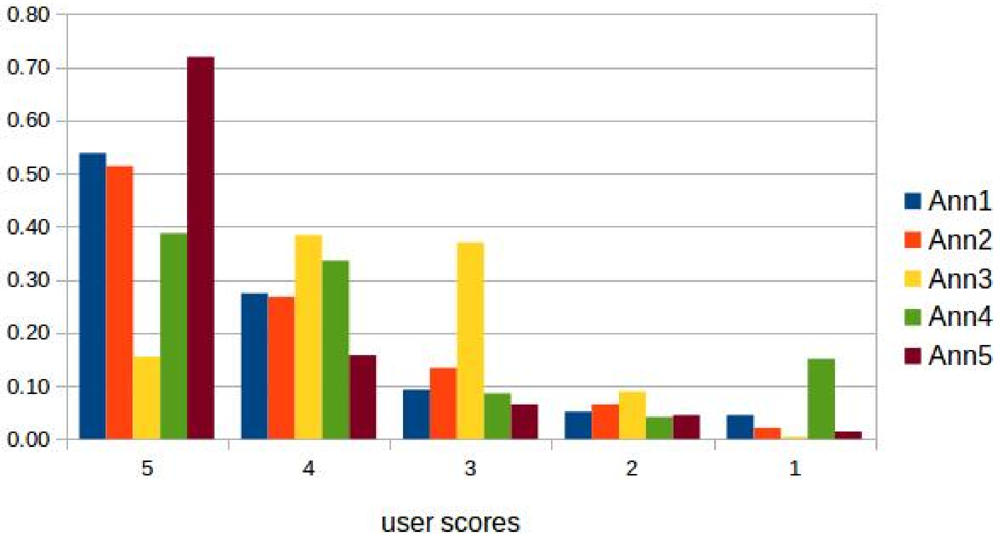

Figure 12: Distribution of scores for uncertainty when annotating events from individual passages on a 1-5 scale

In more detail, the agreement percentages per annotator pair are presented in Table 4. We can see that while the agreement percentage for each pair ranges from 55% (annotators 1 & 5) to 24% (annotators 4 & 5) for most of the disagreements the scores differ by just one point in the grading scale. The percentage of disagreement that exceeds 1 point in the 1-5 scale is presented in Table 5 and ranges between 13% and 37%, while differences greater than 2 points in the scale account for only 1% to 13% of the pairwise disagreement (Table 6). Still, since the annotators were presented with the same sentences, training and annotation guidelines, the deviation in the scoring shows non-uniform perception of the influence of uncertainty expressions over scientific statements. This highlights not only the complexity of the problem, but also hints at the fact that uncertainty scoring might have to be adapted for different users and use-cases.

[10] We note that the annotators for this use-case are different to the ones who carried out the annotations presented in Section 4.2.1 of the main manuscript. Since both tasks presented the users with the same interaction/sentences, we have decided to use a different set of users, in order to avoid biased choices due to having previously seen the decision of the system on the same sentence.

Table 4: Inter-annotator agreement for each annotator pair

	A1	A2	A3	A4	A5
A1	1	0.48	0.33	0.33	0.55
A2	0.48	1	0.36	0.28	0.49
A3	0.33	0.36	1	0.24	0.24
A4	0.33	0.28	0.24	1	0.37
A5	0.55	0.49	0.24	0.37	1

Table 5: Ratio of disagreement greater that 1 point in the 1-5 scale for each annotator pair

	A1	A2	A3	A4	A5
A1	1	0.13	0.24	0.28	0.08
A2	0.13	1	0.19	0.30	0.13
A3	0.24	0.19	1	0.37	0.31
A4	0.28	0.30	0.37	1	0.30
A5	0.08	0.13	0.31	0.30	1

Table 6: Ratio of disagreement greater that 2 points in the 1-5 scale for each annotator pair

	A1	A2	A3	A4	A5
A1	1	0.02	0.01	0.17	0.02
A2	0.02	1	0.01	0.18	0.03
A3	0.01	0.01	1	0.09	0.04
A4	0.17	0.18	0.09	1	0.17
A5	0.02	0.03	0.04	0.17	1

In order to compare with the annotations of our tool, we calculated Precision, Recall and F-score performance for each different uncertainty thresholds in the 1–5 scale with a 0.1 interval. In other words, starting from the highest point in the scale (5) as threshold, we calculate the performance assuming that any event scored with a value smaller than this, should be considered uncertain. We calculated the performance comparing against the mean average score, calculated by the 5 individual scores given by each annotator for each event. The results are presented in Figure 13, for values in [5,1.4], as 5 was the maximum and 1.4 the minimum in the mean average of scores.

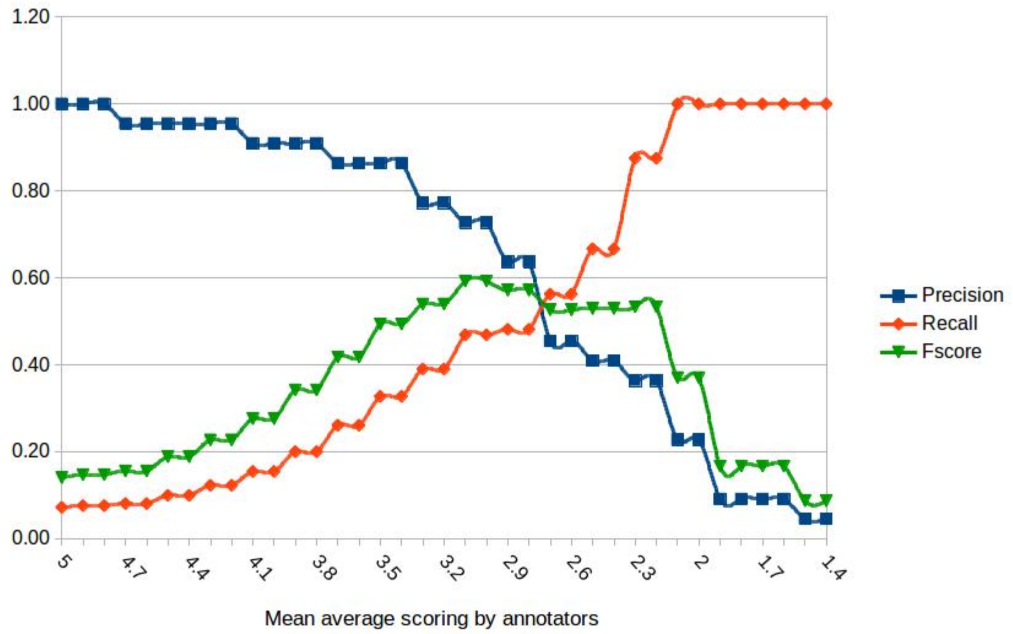

Figure 13: Performance in terms of precision, recall and F-score, depending on the selection of the mean average score to be the threshold of uncertainty (i.e., the value below which all scored events must be considered uncertain)

As expected, recall increases, while precision drops as the threshold moves towards smaller values, rendering the boundaries for uncertainty stricter. It is interesting to notice that for very strict uncertainty boundaries (i.e., with threshold being in $[2.1, 1.4]$ recall reaches 1.00, in other words, our tool marks as uncertain all the events for which users agree in very low uncertainty values (1-2). Similarly no events were marked as uncertain for the cases where the mean average score was greater than 4.8. The threshold values for which F-score peaks (reaching 0.60) are between 3 and 3.1, slightly lower than the corresponding value in the Ras-Melanoma experiment (3.5).

Focussing at the scores attributed to the pathway interactions, we can see the score distribution for each annotator in Figure 14. We can observe that much like the Ras-melanoma case, the perception of uncertainty

over the model interactions seems to follow the same pattern for the perception of uncertainty over the events extracted from evidence sentences for each user.

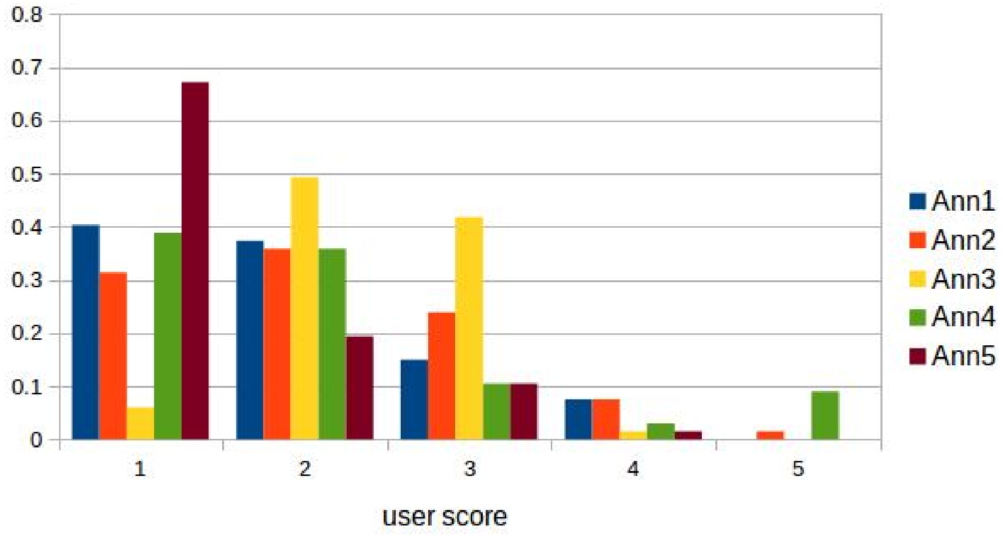

Figure 14: Distribution of scores for uncertainty when annotating interactions based on events from related evidence passages on a 1-5 scale

We evaluate the subjective logic fusion as described in the main manuscript for the Ras-melanoma use-case. In more detail, we firstly consider the results of our system under the following assumptions: (1) α is set to 0.5 for all cases, (2) an event $e1$ that maps to interaction x and is identified as uncertain, constitutes an opinion with $b_x^{e1} = 0.5, u_x^{e1} = 0.5$ and (3) an event $e2$ that maps to interaction x and is identified as certain has $b_x^{e2} = 1, u_x^{e2} = 0$. Additionally, we project the 1-5 scoring to a (0,1] scale by dividing by 5. Thus, we can use Equations 5 and 6 in the main manuscript to calculate the score of the interaction x and then compare it to the scores given by the annotators. We then calculate the absolute difference between the score of the system and the average score given by the annotators. The results are shown in Table 7. In the same table, we compare to the score that would be calculated if we were to estimate each annotator's score for the interaction by applying the Equation 7 of the main manuscript to the scores they attributed to the individual events mapped to that interaction. In other words, we calculated for each annotator the average absolute difference between the score given to the interaction by him/her and the fused score calculated with Equation 7 using the scores given by the same annotator for each event mapped to the interaction.

Table 7: Results for the interaction scoring on the Ras-melanoma network

	Ann1	Ann2	Ann3	Ann4	Ann5	Mean Avg (Ann 1-5)	System Prediction
Mean average difference	0.09	0.12	0.09	0.10	0.08	0.10	0.17
Standard Deviation	0.10	0.11	0.07	0.13	0.07	0.10	0.09

We can observe that the score predictions when using the scores given to the events by the annotators are very close to the actual scores attributed by them, since with the mean average difference being 0.10. Considering the fact that the scoring scale was in [1,5] this means that if we were to project this deviation to the initial scale, it would be less than one point in the scale. Similarly to the Ras-Melanoma use-case described in the main manuscript (Section 4.2), the score given by the system deviates slightly more (but is still marginally less than 1 point if projected to the original 1-5 scale). It has to be highlighted that such a deviation is partly due to the fact that our system makes use of binary classification of uncertainty.

Hence, the results from this re-annotation effort seem to further strengthen our claim in the main manuscript that subjective logic seems to provide a good approximation of the score and way users assess uncertainty based on a series of statements by different authors.

References

Alias-i (2008). Lingpipe 4.1.0. http://alias-i.com/lingpipe. (accessed November 2016).

Brin, S., Motwani, R., Ullman, J. D., and Tsur, S. (1997). Dynamic itemset counting and implication rules for market basket data. *Proceedings of ACM SIGMOD International Conference on Management of Data*, 255–264.

Cohen, J. and Jacob (1968). Weighted kappa: Nominal scale agreement provision for scaled disagreement or partial credit. *Psychological Bulletin*, **70**, 213–220.

Hall, M., Frank, E., Holmes, G., Pfahringer, B., Reutemann, P., and Witten, I. H. (2009). The WEKA data mining software. *ACM SIGKDD Explorations Newsletter*, **11**(1), 10.

Jessop, D. M., Adams, S. E., Willighagen, E. L., Hawizy, L., and Murray-Rust, P. (2011). Oscar4: a flexible architecture for chemical text-mining. *Journal of cheminformatics*, **3**, 41–53.

Matsuzaki, T. and Tsujii, J. (2008). Comparative parser performance analysis across grammar frameworks through automatic tree conversion using synchronous grammars. *Proceedings of the 22nd ACL*, **1**, 545–552.

Miwa, M., Thompson, P., McNaught, J., Kell, D. B., and Ananiadou, S. (2012). Extracting semantically enriched events from biomedical literature. *BMC bioinformatics*, **13**, 108–132.

Miwa, M., Pyysalo, S., Ohta, T., and Ananiadou, S. (2013). Wide coverage biomedical event extraction using multiple partially overlapping corpora. *BMC bioinformatics*, **14**, 175.

Nawaz, R., Thompson, P., and Ananiadou, S. (2013). Negated bio-events: analysis and identification. *BMC Bioinformatics*, **14**, 1–21.

Nédellec, C., Bossy, R., Kim, J.-D., Kim, J.-J., Ohta, T., Pyysalo, S., and Zweigenbaum, P. (2013). Overview of BioNLP shared task 2013. *Proceedings of BioNLP 2013*, 1–7.

Nikitin, A., Egorov, S., Daraselia, N., and Mazo, I. (2003). Pathway studio–the analysis and navigation of molecular networks. *Bioinformatics*, **19**(16), 2155–7.

Piatetsky-Shapiro, G. (1991). Discovery, analysis and presentation of strong rules. *Knowledge discovery in databases*, 229–248.

Rak, R., Rowley, A., Black, W., and Ananiadou, S. (2012). Argo: an integrative, interactive, text mining-based workbench supporting curation. *Database*, 1–10.

Smyth, P. (1991). Rule induction using information theory. *Knowledge discovery in databases*, 159–176.

Stenetorp, P., Pyysalo, S., Topić, G., Ohta, T., Ananiadou, S., and Tsujii, J. (2012). Brat: a web-based tool for nlp-assisted text annotation. *Proceedings of Demonstrations at 13th EACL*, 102–107.

Tan, P.-N., Kumar, V., and Srivastava, J. (2004). Selecting the right objective measure for association analysis. *Information Systems*, **29**(4), 293–313.

Thompson, P., Nawaz, R., McNaught, J., and Ananiadou, S. (2016). Enriching news events with meta-knowledge information. *LREC*, 1–30.

Tsuruoka, Y., Tateishi, Y., Kim, J.-D., Ohta, T., McNaught, J., Ananiadou, S., and Tsujii, J. (2005). Developing a robust part-of-speech tagger for biomedical text. In *Panhellenic Conference on Informatics*, 382–392.

Zerva, C. and Ananiadou, S. (2015). Event extraction in pieces: Tackling the partial event identification problem on unseen corpora. *ACL-IJCNLP 2015*, 31–41.
